# Mechanisms underlying cognitive deficits in a mouse model for Costello Syndrome are distinct from other RASopathy mouse models

**DOI:** 10.1038/s41598-017-01218-0

**Published:** 2017-04-28

**Authors:** Jadwiga Schreiber, Laura-Anne Grimbergen, Iris Overwater, Thijs van der Vaart, Jeffrey Stedehouder, Alberto J. Schuhmacher, Carmen Guerra, Steven A. Kushner, Dick Jaarsma, Ype Elgersma

**Affiliations:** 1 Department of Neuroscience, Erasmus Medical Centre Rotterdam, 3015 CN Rotterdam, The Netherlands; 2 ENCORE Expertise Center for Neurodevelopmental Disorders, Erasmus Medical Centre Rotterdam, 3015 CN Rotterdam, The Netherlands; 3 Department of Psychiatry Erasmus Medical Centre Rotterdam, 3015 CN Rotterdam, The Netherlands; 4 Molecular Oncology, Centro Nacional de Investigaciones Oncológicas (CNIO), Madrid, Spain

## Abstract

RASopathies, characterized by germline mutations in genes encoding proteins of the RAS-ERK signaling pathway, show overlapping phenotypes, which manifest themselves with a varying severity of intellectual disability. However, it is unclear to what extent they share the same downstream pathophysiology that underlies the cognitive deficits. Costello syndrome (CS) is a rare RASopathy caused by activating mutations in the *HRAS* gene. Here we investigated the mechanisms underlying the cognitive deficits of *HRas* ^*G12V*/*G12V*^ mice. *HRas* ^*G12V/G12V*^ mice showed robust upregulation of ERK signaling, neuronal hypertrophy, increased brain volume, spatial learning deficits, and impaired mGluR-dependent long-term depression (LTD). In contrast, long-term potentiation (LTP), which is affected in other RASopathy mouse models was unaffected. Treatment with lovastatin, a HMG-CoA-Reductase inhibitor which has been shown to rescue the behavioral phenotypes of mouse models of NF1 and Noonan syndrome, was unable to restore ERK signaling and the cognitive deficits of *HRas* ^*G12V/G12V*^ mice. Administration of a potent mitogen-activated protein kinase (MEK) inhibitor rescued the ERK upregulation and the mGluR-LTD deficit of *HRas* ^*G12V/G12V*^ mice, but failed to rescue the cognitive deficits. Taken together, this study indicates that the fundamental molecular and cellular mechanisms underlying the cognitive aspects of different RASopathies are remarkably distinct, and may require disease specific treatments.

## Introduction

In the last decade, several neurodevelopmental syndromes have been identified with germline mutations within key components of the highly conserved RAS (rat sarcoma viral oncogene homolog)-ERK (extracellular signal regulated kinase) signaling pathway^1–3^. These syndromes, including neurofibromatosis Type I (NF1), Noonan syndrome, LEOPARD syndrome, cardio-facio-cutaneous (CFC) syndrome, Costello syndrome, and Legius syndrome show significant phenotypic overlap and are referred to as RASopathies, the most common group of neurodevelopmental syndromes, affecting approximately 1 in 1,000 individuals^4^. RASopathies patients typically present a combination of facial and skin abnormalities, heart defects, a predisposition to specific cancers, and developmental delay including central nervous system (CNS) abnormalities, cognitive dysfunction, and behavioral impairments.

The RAS-ERK pathway operates as a functional hub that integrates extracellular signals through cell surface receptor activation (e.g., tyrosine kinases receptors (RTKs), and transmits these signals intracellularly^5, 6^. Upon activation, RAS, a small guanosine nucleotide-bound GTPase, switches to an active guanosine triphosphate (GTP)-bound form and activates downstream targets of the pathway. RAS-GTP inactivates rapidly by conversion to RAS-guanosine diphosphate (RAS-GDP) through its intrinsic GTPase^7^. This process can be accelerated by GTPase activating proteins (GAPs) such as Neurofibromin 1 (NF1).

In mitotic cells, the RAS-ERK pathway regulates fundamental processes such as the cell cycle, cellular growth, differentiation, and senescence, all of which are critical to normal development. Not surprisingly, the RAS-ERK pathway has been studied extensively in the context of oncogenesis. However, involvement of the RAS-ERK pathway in a number of neurological syndromes has refocused attention to the function of these proteins in post-mitotic neuronal cell types^3^. Studies of several mouse models of RASophaties, including NF1, NS, and Legius syndrome, have demonstrated that dysregulation of the RAS-ERK pathway causes behavioral and learning deficits, altered synaptic plasticity, and structural brain abnormalities^8–11^. Moreover, the﻿se studies demonstrated the potential of inhibitors of the RAS-ERK pathway as a putative therapy for the cognitive deficits^8–10^.

Costello syndrome (CS) is a rare RASopathy, with a estimated prevalence of 1 in 1.25 million people^12, 13^. CS is caused by gain-of-function missense mutations in the *HRAS* gene, of which the substitution of Glycine to Serine (G12S) at codon 12 in exon 1 is the most prevalent, constituting 80% of CS cases^7, 14, 15^. The G12S mutation reduces the intrinsic GTPase activity of the HRAS protein, with a concomitant increase in binding affinity and activation of its downstream targets including ERK and PI-3 kinase^6^. An *HRAS* ^*G12V/G12V*^ mouse model of CS has been generated which displays several of the phenotypic abnormalities observed in CS patients, including facial dysmorphia and cognitive impairment^16, 17^. However, the cellular mechanism underlying these deficits as well as the ability to reverse them has not yet been studied.

Here we show that *HRAS* ^*G12V/G12V*^ mice, present with strong hyperactivation of the ERK signaling pathway, neuronal hypertrophy with concomitantly increased brain weight, and a spatial learning deficit in the Morris water maze test. At the physiological level we observed normal long-term potentiation (LTP) but impaired mGluR-mediated long-term depression (LTD). Treatment with lovastatin, a drug that rescues learning deficits in NF1 and Noonan syndrome mouse models, had no effect on pERK levels and did not rescue the learning deficits of *HRas* ^*G12V/G12V*^ mice. Notably however, administration of a mitogen-activated protein kinase kinase (MEK) inhibitor normalized the ERK upregulation and rescued the mGluR-LTD deficit of *HRas* ^*G12V/G12V*^ mice, but the cognitive deficits remained persistent. Our results indicate that although all RASopathies are characterized by increased RAS-ERK signaling, their downstream effectors and therapeutic targets appear to be quite distinct.

## Results

### Increased RAS-ERK signaling in *HRas*^*G12V/G12V*^ mice

**Figure 1:**
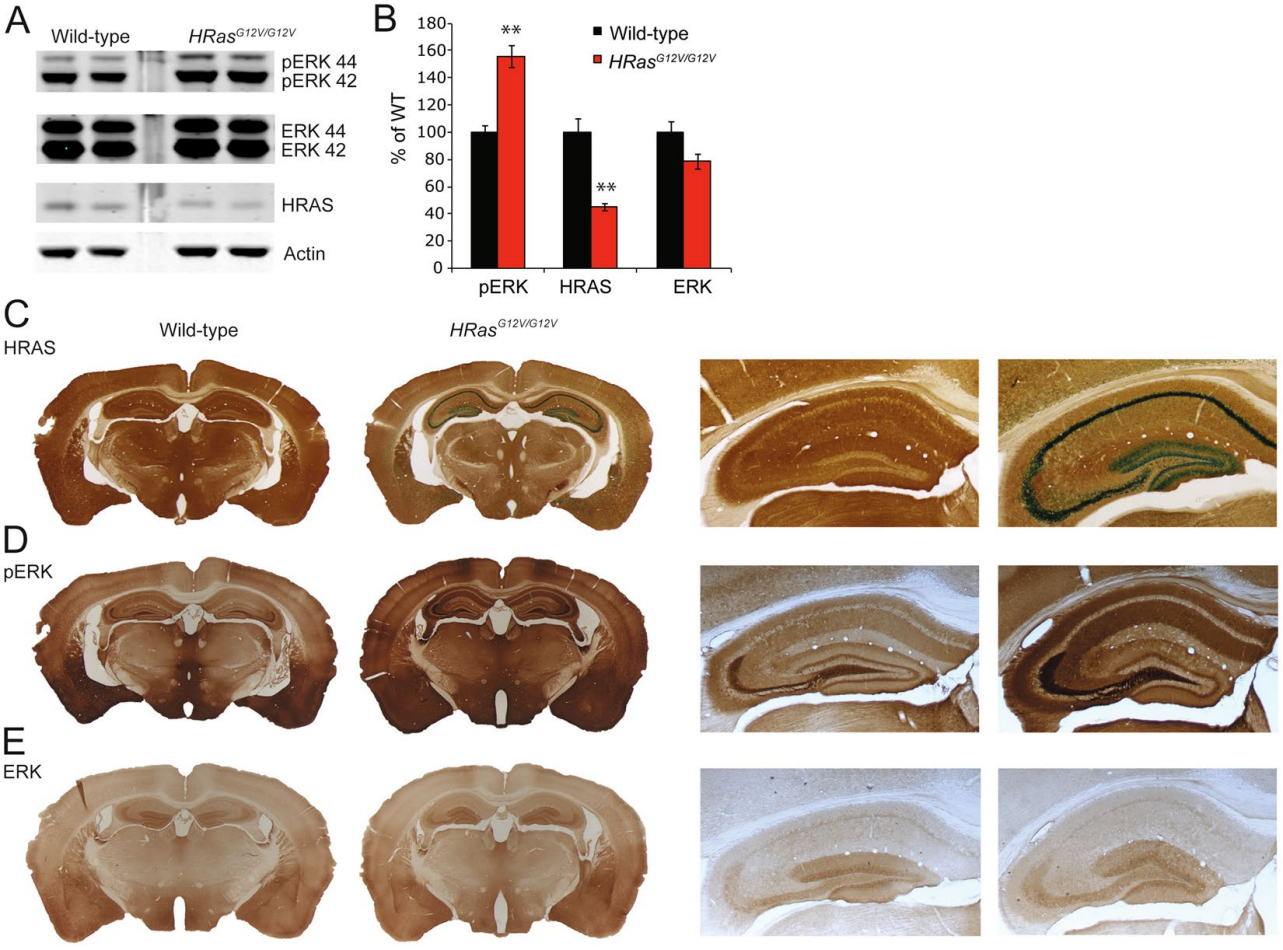
Hyperactivity of RAS-ERK signaling in *HRas* ^*G12V/G12V*^ mice. (**A**) Western blots of hippocampal lysates taken from wild-type and *HRas* ^*G12V/G12V*^ mice using antibodies for HRAS, pERK 42/44 (pERK), ERK 42/44 (ERK) and actin. (**B**) Quantification of Western blots showing a significant increase in pERK (*t*_*8*_ = −5.92, *P* < 0.01) and a significant decrease in expression of HRAS protein in *HRas* ^*G12V/G12V*^ mice compared to their wild-type littermates (*t*_*8*_ = 5.34, *P* < 0.01). Data are presented as mean ± SEM of percentage of WT level set as 100% from 5 mice per genotype (n = 5/genotype). Statistical test: unpaired two-tailed t-test, *P < 0.05, **P < 0.01. (**C**) Visualisation of β-galactosidase activity of the IRES–β-geo cassette integrated into the mutated *HRAS* gene (LacZ; blue) combined with an HRAS staining (DAB; brown) indicating successful recombination and expression of HRAS^G12V^, however lower amounts of HRAS protein are seen in *HRas* ^*G12V/G12V*^ mice compared to WT littermates. Right panel: Zoomed-in image of hippocampus. (**D,E**) DAB staining using pERK 42/44 and ERK 42/44 antibodies revealed increased levels of ERK phosphorylation in *HRas* ^*G12V/G12V*^ mouse brain slices compared to WT (**D**), but no difference in the level of expression and distribution of total ERK between genotypes (**E**).

To better understand the role of HRAS protein in neuronal functioning, we studied a mouse model for Costello syndrome (CS)^16^. This mouse carries a germline targeted point mutation introduced into the *HRAS* gene: glycine (G) to valine (V) at amino acid 12 (G12V), leading to expression of a constitutively active HRAS protein (from here referred to as HRAS^G12V^ protein or *HRas* ^*G12V/G12V*^ mice). The G12V mutation has been found in a number of CS patients and is associated with very severe disease manifestations^14, 18, 19^. As described previously, mutant *HRas* ^*G12V/G12V*^ mice were born at the expected Mendelian ratio, were fertile, survived at rates comparable to those of their wild-type littermates, and displayed several of the phenotypic abnormalities observed in CS patients, including facial dysmorphia^16, 17^. In examining protein expression, we observed a 50% decrease in HRAS protein in hippocampal lysates of *HRas* ^*G12V/G12V*^ mice (*t*_*8*_ = 5.34, *P* < 0.01; Fig. 1A,B), which was confirmed by immunohistochemistry (Fig. 1C). Hence, the level of HRAS^G12V^ protein in homozygous *HRas* ^*G12V/G12V*^ mice might resemble the HRAS^G12V^ protein level of heterozygous patients.

To evaluate whether constitutively active HRAS leads to hyperactivation of the RAS-ERK pathway, we assessed the level of ERK phosphorylation (pERK) in hippocampal lysates of adult *HRas* ^*G12V/G12V*^ mice. Immunoblotting revealed a significant increase in phosphorylated ERK isoforms ERK1 (pERK 44) and ERK2 (pERK 42) (*t*_*8*_ = −5.92, *P* < 0.01, whereas total ERK was similar between the genotypes (Fig. 1A,B). In accordance with this result, immunohistochemistry revealed increased amounts of pERK throughout the *HRas* ^*G12V/G12V*^ brain, particularly in the hippocampus and cortex (Fig. 1D,E).

### Hypertrophy of brain and pyramidal neurons in *HRas*^*G12V/G12V*^ mice

**Figure 2:**
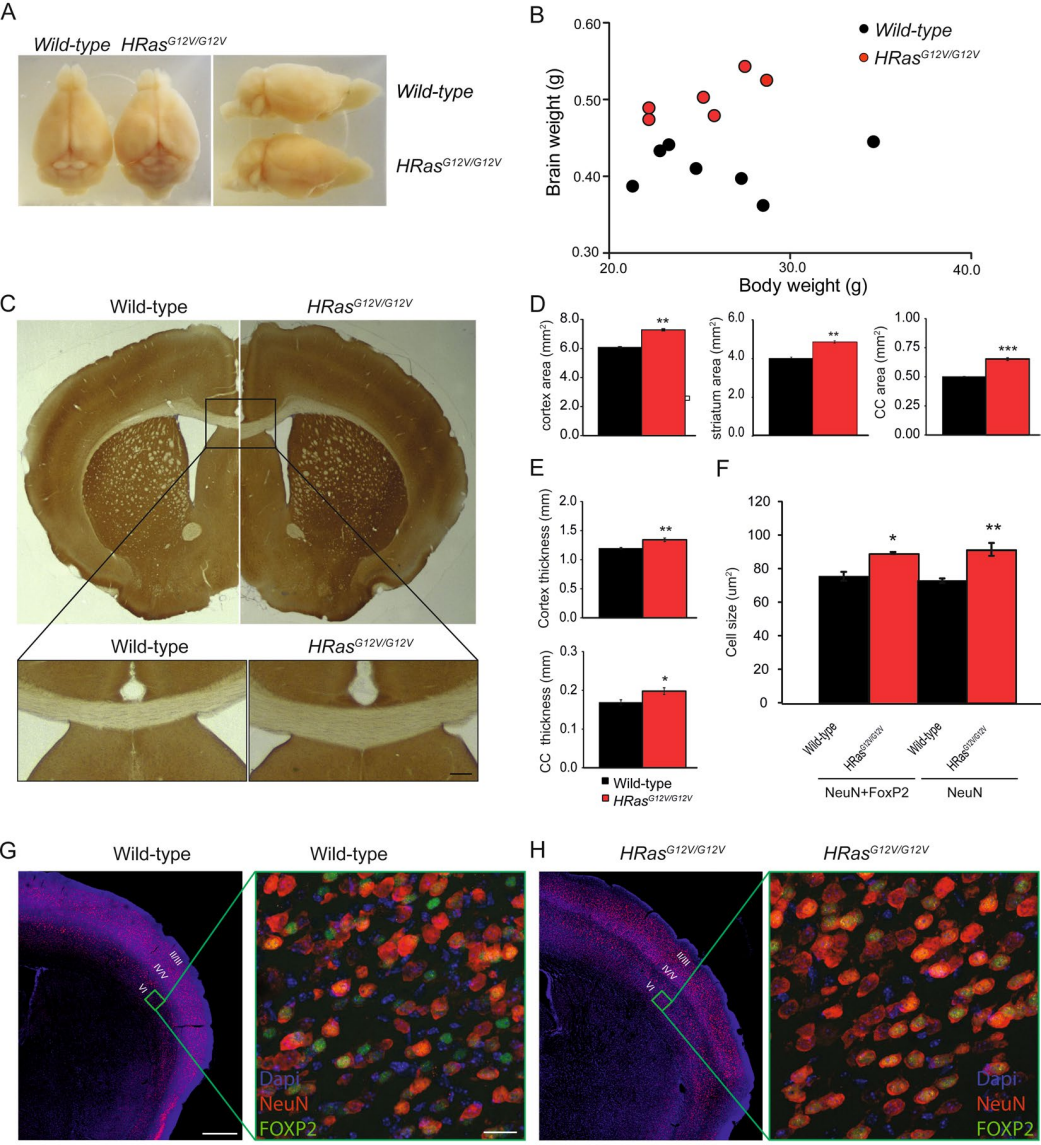
Hypertrophy of brain and pyramidal neurons in *HRas* ^*G12V/G12V*^ mice. (**A**) Overview images of brains dissected from *HRas* ^*G12V/G12V*^ mice and wild-type littermates, indicating the increased brain weight of *HRas* ^*G12V/G12V*^ mice. (**B**) *HRas* ^*G12V/G12V*^ mice have significantly increased brain weight (effect of genotype: *F*_*1,11*_ = 31.59, P < 0.01) but show no difference in body weight (effect of genotype: *F*_*1,11*_ = 0.15, P = 0.71). Data are presented as individual values of brain vs. body weight from wild-type n = 7 mice, *HRas* ^*G12V/G12V*^ n = 6 mice. Statistical test: one-way MANOVA. (**C**) Coronal cross-section comparison between *HRas* ^*G12V/G12V*^ and wild-type mice. Upper panel: As an example, the measurements of surface area of corpus callosum (CC) are indicated by yellow lines; cortical thickness was measured as indicated by the black line in the left hemisphere. Lower panel: Zoomed-in images of CC in the midline of the section of WT and *HRas* ^*G12V/G12V*^ mice represent thicker CC in *HRas* ^*G12V/G12V*^ mice. (**D,E**) Quantification of the area measurements revealed significantly bigger surface areas of cortex (*t*_*6*_ = −13.05, P < 0.01), striatum (t6 = −10.27, P < 0.01) and corpus callosum (*t*_*6*_ = −12.49, P < 0.01) (**D**) as well as a thicker cortex (*t*_*6*_ = −4.05, P < 0.01) and CC (*t*_*6*_ = −2.56, P < 0.05) (**E**). (**F**) Quantification of cell size revealed a significantly bigger soma size of NeuN- and FoxP2-positive cells as well as in NeuN-positive pyramidal neurons in *HRas* ^*G12V/G12V*^ mice (for NeuN-positive cells *t*_*6*_ = −3.58, P < 0.05; for NeuN- and FoxP2-positive cells *t*_*6*_ = −4.76, P < 0.01). (**G,H**) Images of coronal slices of the cortex stained for DAPI (blue) and NeuN (red) in wild-type (**G**) and *HRas* ^*G12V/G12V*^ (**H**) mice. The green square indicates layer VI of the primary somatosensory cortex barrel field (S1BF), an area chosen for cell size measurements (zoomed-in images). Zoomed-in pictures of layer VI of S1BF illustrates pyramidal neurons stained using DAPI (blue), NeuN (red) and FOXP2 (green; a marker for deep cortical layers in wild-type (**G**) and *HRas* ^*G12V/G12V*^ (**H**) mice. For (**C–H**) Data are presented as mean ± SEM from 4 hemispheres/2 mice per genotype. Statistical test: unpaired two-tailed *t*-test, *P < 0.05, **P < 0.01, ***P < 0.001. Scale bars G: 500 µm (left), (**G**) 20 µm (right).

Similar to the previous descriptions of the CS mouse model, we observed that most *HRas* ^*G12V/G12V*^ mice could be clearly distinguished from their wild-type (WT) littermates by their blunt nose and prominent forebrain^16, 17^. After careful dissection of the brain from the skull, we observed a difference in the overall shape of the brain of *HRas* ^*G12V/G12V*^ mice (Fig. 2A). Brain weight was increased by 22% in *HRas* ^*G12V/G12V*^ mice (*F*_*1,11*_ = 31.59, P < 0.01; Fig. 2B), while total body weight was unchanged (*F*_*1,11*_ = 0.15, P = 0.71). Measurement of the surface area of several brain regions on level-matched coronal brain sections revealed that *HRas* ^*G12V/G12V*^ mice had a significantly larger surface area of cortex (*t*_*6*_ = −13,05, P < 0.01), striatum (*t*_*6*_ = −10.27, P < 0.01), and corpus callosum (*t*_*6*_ = −12.49, P < 0.01, Fig. 2D). Moreover, both the cortex (*t*_*6*_ = −4.05, P < 0.01) and corpus callosum (*t*_*6*_ = −2.56, P < 0.05) were significantly thicker in *HRas* ^*G12V/G12V*^ mice (Fig. 2C,E). In addition, analysis of neuronal cell size was performed in the deep lamina of the S1 somatosensory cortex in transverse sections stained for DAPI, NeuN and FOXP2, which is expressed by a subset of neurons in layer V and VI. The diameter of both NeuN+FOXP2 negative and NeuN+FOXP2 positive neurons was significantly larger in *HRas* ^*G12V/G12V*^ mice (upper-layer neurons: *t*_*6*_ = −3.58, P < 0.05; deep-layer neurons *t*_*6*_ = −4.76, P < 0.01; Fig. 2F–H). These results are in agreement with previous observations from a transgenic *HRas* ^*G12V*^ mouse model, which showed increased brain volume and larger pyramidal cells in layers V-VI of the motor and somatosensory cortex in adult mice^20, 21^.

### Learning impairment in *HRas*^*G12V/G12V*^ mice in the Morris water maze (MWM)

**Figure 3:**
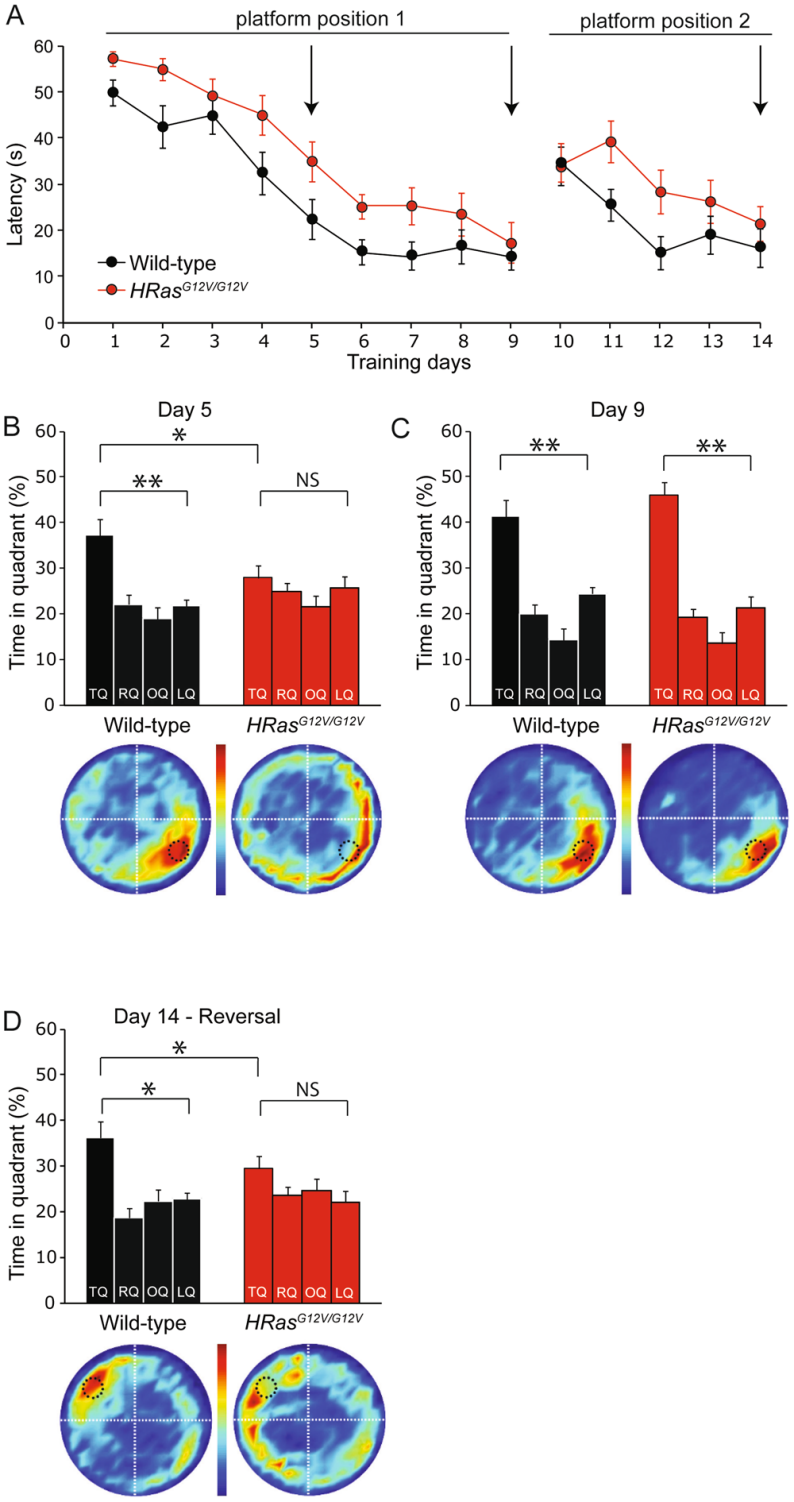
Learning impairment in *HRas* ^*G12V/G12V*^ mice in the Morris water maze (MWM). (**A**) Average latency to find the platform for each day of training. The first 9 days represent hidden platform MWM training for the initial platform position (platform position 1). On day 10, the reversal MWM protocol was started by changing the platform position (platform position 2). A decrease in the latencies of both genotypes can be observed, however on average *HRas* ^*G12V/G12V*^ mice took significantly more time to find the platform (between subject comparison day 1–9: *F*_1,30_ = 12.78, *P* < 0.01; day 10–14: *F*_1,15_ = 6.53, *P* < *0.05*. (**B**) When spatial memory was assessed in a probe trial after 5 days of training, wild-type mice preferentially searched in the target quadrant (TQ, black bar) where the submerged platform had been during the training sessions (TQ vs. other quadrants: P < 0.01), whereas *HRas* ^*G12V/G12V*^ mice did not show a preference for the target quadrant (P = 0.15); between subject comparison: effect of genotype, P < 0.05). TQ = target quadrant, RQ = adjacent right quadrant, LQ = adjacent left quadrant, OQ = opposite quadrant. (**C**) A probe trial after four additional days of training showed that *HRas* ^*G12V/G12V*^ mice catch up with their WT littermates (TQ vs. other quadrants: *HRas* ^*G12V/G12V*^: P < 0.01, WT: P < 0.01; between subject comparison: effect of genotype P = 0.79). Heat plots beneath the graphs provide a visual representation of all search tracks, in which the color indicates the mean time spent at a certain position in the pool. (**D**) After hidden-platform training mice were re-trained in a reversal MWM task. After 5 days of training for a new platform position (day 10–14; platform position 2), wild-type mice but not *HRas* ^*G12V/G12V*^ animals showed a preference for the TQ (TQ vs. other quadrants wild-type*:* P < 0.01; TQ vs. OQ *HRas* ^*G12V/G12V*^: P = 0.14; between subject comparison: effect of genotype P = 0.17). Data are presented as mean ± SEM from wild-type n = 17 mice, *HRas* ^*G12V/G12V*^ n = 15 mice (for **A,B**) and from wild-type n = 10 mice, *HRas* ^*G12V/G12V*^ n = 8 mice (for **C**). Statistical test: repeated measure one-way MANOVA, paired two-tailed *t*-test and one-way MANOVA. Arrows in **A** indicate day of probe trials.

Learning deficits are among the most frequent cognitive limitations observed in CS patients^22^ as w﻿ell as other R﻿ASopathies^3^, and are also observed in mouse models of CS, NF1, NS, and Legius syndrome. In particular, impairments of hippocampus-dependent spatial learning in the Morris water maze (MWM) have been reported in every RASopathy mouse model examined thus far (Table 1). During the MWM training, mice learn to use visual cues positioned outside a pool to find a hidden escape platform submerged beneath the surface of the opaque water. Previously, it has been shown that *HRas* ^*G12V/G12V*^ mice in the mixed 129-C57BL/6 background showed a learning deficit in the MWM test^17^. However, it has been frequently been observed that behavioral phenotypes are lost upon backcrossing. In fact expression of NF1, the most important regulator of RAS, is 30% lower in animals on the 129 strain compared to the animals on the C57BL/6 strain^23^, and the learning deficits in NF1 mice are exclusively observed in the mixed (F1) 129-C57BL/6 background^8, 24, 25^. Hence, to determine whether the MWM phenotype of *HRas* ^*G12V/G12V*^ mice is still present upon backcrossing the mutants for over 20 generations in the C57BL/6 background, we repeated the MWM experiment. We observed that both genotypes showed a reduction in their latency to find the hidden platform, but *HRas* ^*G12V/G12V*^ mice exhibited significantly longer latencies compared to wild-type mice (between subject comparison day 1–9: *F*_*1,30*_ = 12.78, *P* < 0.01; day 10–14: *F*_*1,15*_ = 6.53, *P* < 0.05; Fig. 3A). There were no significant differences in swimming speed (between subject comparison, effect of genotype: *F*_*1,30*_ = 0.28, P = 0.60) or thigmotaxis (*F*_*1,30*_ = 190, P = 0.18).

**Table 1:** Overview and comparison of phenotypes and treatments in mouse models for four different RASopathies.

Phenotype	Mouse Model	Costello Syndrome (CS)	Neurofibromatosis Type 1 (NF1)	Noonan Syndrome (NS)^10^	Legius syndrome (LS)^11^
Gene		*HRAS*	*NF1*	*PTPN11*	*SPRED1*
Mutation		*HRAS* ^*G12V/G12V*^	*NF* ^+/−^	*Ptpn11* ^*D61G*/+^	*SPRED1* ^−/−^
pERK level		Strongly increased^#^	Moderately increased^8, 24, 31^	Moderately-strongly increased	Normal-slightly increased
**Behavior**					
Morris Water Maze		Impaired^#, 17^	Impaired^8, 24, 25, 31^	Impaired	Impaired
Contextual fear conditioning		Normal^#, 1, 17^	Impaired^8, 24^	Impaired	
Rotarod		Normal^#, 17^	Impaired^25, 54^		Normal
**Neuronal function**					
LTP (TBS induced)		Normal^#^﻿	Impaired ↓^8, 24, 25, 31^	Impaired ↓	Impaired ↓
LTD		mGluR-LTD ↓^#^			LFS-LTD ↑
Inhibitory function (s/m/eIPSCs)^*^		Normal^#^	Increased^8, 24, 25^	Normal	
Excitatory function (s/mEPSCs)		Normal^#^	Normal^24, 25^	Increased	
I/O at strong stimulation		Normal^#^	Impaired^8, 24^	Normal	Impaired
Short term/PPF		Normal^#^	Normal^8, 25^	Normal	↑
I_*h*_ current		Normal^25^	↓^25^		
**Brain morphology**					
Craniofacial features		Abnormal^#, 2^	Normal^8, 25^	Abnormal^52^	
Gross brain morphology		Abnormal^#^; Normal^39^, ^**^			
Cell size/Cell number		Increased^#^, ^4^/Increased^39^, ^*^			
**Treatment: Lovastatin**					
Treatment		10 mg/kg, IP, adult mice, 3 days before the first training day, and then daily during behavioral training^#^, ^9, 31^			
pERK level normalized?		No	Yes	Yes	
Behavioral deficit rescued?		No	Yes	Yes	
LTP rescued?		(No phenotype)	Yes	Yes	
**Treatment: MEK-inhibitor**					
Drug		PD0325901^#^	PD0325901^9^	SL327	
Treatment		2 mg/kg, IP, adult mice, 3 days before the first training day, and then daily during behavioral training	5 mg/kg, oral gavage, daily administration to lactating females for the treatment of P0.5– P18 mice	32 mg/kg, IP, adult mice, daily during behavioral training	
pERK level normalized?		Yes	Yes^9^	Yes	
Behavioral deficit rescued?		No		Yes	
Brain morphology rescued?		No	Yes^9^		
Neuronal function rescued?		Yes (mGlur-LTD)^#^ (U0126, 1 µM)	Yes (mIPSC)^24^, (U0126, 10 µM)	Yes (LTP) (SL327, 1 µM)	

Abbreviations: TBS – theta burst stimulation; LFS - low frequency stimulation, ↑ - significant increase; ↓ - significant decrease; Normal - no difference observed; “−” – no rescue observed; “+” – rescue observed; LTD – long-term depression; LTP – long-term potentiation; I/O – input/output; pERK – phosphorylated ERK; IP – Intra peritoneal injection; s-, m-, e E/IPSCs – spontaneous-, miniature- or evoked- excitatory (E)/inhibitory (I) postsynaptic currents; * - mIPSCs recordings in high KCl concentration (12,5 mM); ** - young animals, E14-P7; ^﻿#﻿^- current study.

After 5 days of hidden platform training, the platform was removed and mice were subjected to a probe trial to test spatial memory. Wild-type mice demonstrated a focused search pattern with the majority of their time spent in close proximity to the trained position of the platform (target quadrant (TQ) versus other quadrants; t_*16*_ = −3.46, P < 0.01). In contrast, *HRas* ^*G12V/G12V*^ mice showed no significant preference for the target quadrant compared to the other three quadrants and were significantly different from wild-type mice (TQ vs. other quadrants: *t*_*14*_ = −1.05, P = 0.16; between subject comparison *F*_*1,30*_ = 4.20, P < 0.05; Fig. 3B). After 4 additional days of training, mutants and wild-type mice reached the platform at comparable times (Fig. 3A), after which a probe trial confirmed that both wild-type and *HRas* ^*G12V/G12V*^ animals had learned to find the position of the platform and showed selective spatial searching (TQ vs. OQ, *HRas* ^*G12V/G12V*^: t_*14*_ = −4.64, P < 0.01; WT: t_16_ = −5,47, P < 0.01; between subject comparison, effect of genotype: *F*_*1,30*_ = 0.42, P = 0.79; Fig. 3C). These results indicate that *HRas* ^*G12V/G12V*^ mutant mice in the C57BL/6 background have a spatial learning impairment that can be overcome with additional training.

In order to assess the cognitive flexibility of *HRas* ^*G12V/G12V*^ mice, the platform was then relocated to the opposite quadrant for 5 additional training days, beginning on day 10. A probe trial on day 14 revealed that wild-type mice had successfully learned the new platform position (TQ vs. OQ: *t*_*9*_ = −3.89, P < 0.01), whereas *HRas* ^*G12V/G12V*^ mice again showed no preference for the target quadrant (TQ vs. OQ: *t*_*7*_ = −1.18, P = 0.14; between subject comparison: *F*_*1,16*_ = 2.01, P = 0.17; Fig. 3A,D). Therefore, we conclude that *HRas* ^*G12V/G12V*^ mice have reduced spatial learning abilities, which may also impair cognitive flexibility (reversal MWM phenotype).

### Baseline synaptic transmission and LTP are unaffected in *HRas*^*G12V/G12V*^ mice

**Figure 4:**
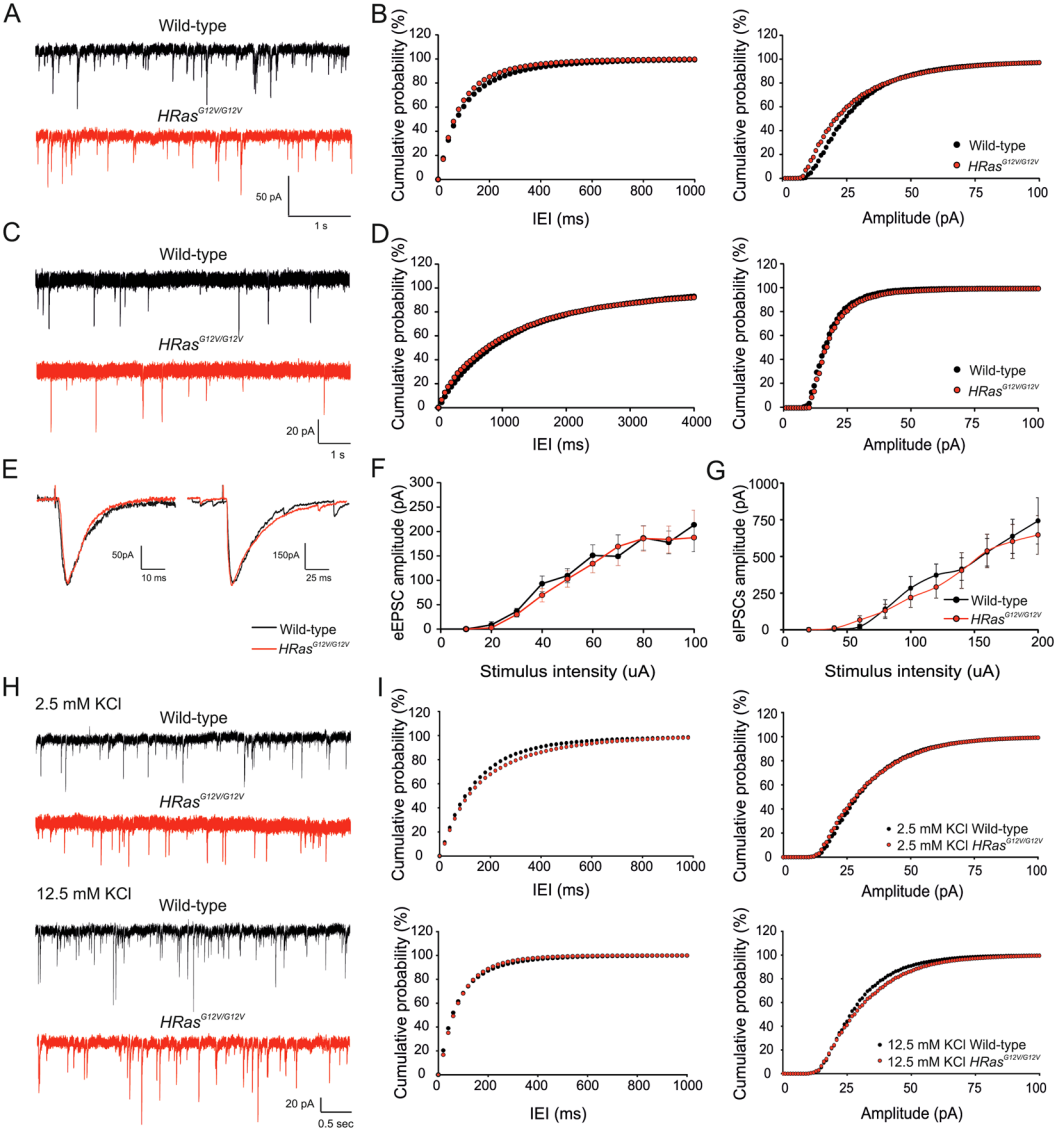
Normal baseline synaptic transmission in wild-type mice. (**A** and **C**) Representative traces of whole-cell patch-clamp recordings of spontaneous inhibitory (sIPSC) (**A**) and excitatory (sEPSC) (**C**) postsynaptic currents in acute hippocampal slices (**B**,**D**) Cumulative distribution of inter-event intervals (IEI; left panels) and amplitude (right panels) of sIPSCs and sEPSCs revealed no significant differences between the genotypes (sIPSCs: frequency: average: *t*_*44*_ = −0.39, P = 0.69, cumulative: P > 0.10; amplitude: average: *t*_*44*_ = 0.38, P = 0.70, cumulative: P > 0.10; wild-type n = 28 cells/7mice, *HRas* ^*G12V/G12V*^ n = 26 cells/5 mice) (sEPSCs: frequency: average: *t*_*44*_ = −0.32, P = 0.75, cumulative: P > 0.10; amplitude: average: *t*_*44*_ = 0.67, P = 0.50, cumulative: P > 0.10; wild-type n = 20 cells/5 mice, *HRas* ^*G12V/G12V*^ n = 26 cells/7 mice). Statistics: two-tailed t-test and Kolmogorov–Smirnov test (K–S) for average and cumulative values, respectively. (**E**–**G**) Evoked IPSCs and EPSCs are not altered in *HRas* ^*G12V/G12V*^ mice. (**E**) Representative traces of evoked EPSC (left) and IPSC (right) of wild-type cells (black trace) and *HRas* ^*G12V/G12V*^ cell (red trace). (**F**,**G**) Input-output curve representing averaged eIPSCs (wild-type n = 18 cells/10 mice, *HRas* ^*G12V/G12V*^ n = 14 cells/8 mice; (**F**) and eEPSCs (wild-type n = 19 cells/9 mice, *HRas* ^*G12V/G12V*^ n = 20 cells/10 mice; **G**) Data are presented as mean ± SEM. Statistical test: repeated measure ANOVA: eEPSCs: F_*1,39*_ = 0.26, P = 0.61; eIPSCs: F_*1,32*_ = 0.01, P = 0.95. (**H,I**) Representative traces of whole-cell patch-clamp recordings of miniature inhibitory postsynaptic currents (mIPSC) in 2.5 and 12.5 mM KCl. A significant increase in the average frequency between 2.5 and 12.5 mM KCl was observed (*F*_*1,49*_ = 22,28, P < 0.01) but not in amplitude (*F*_*1,49*_ = 1,21, *P* = 0.28) nor between genotypes (genotypes: frequency: *F*_*1,49*_ = 1.07, *P* = 0.30, amplitude: *F*_*1,49*_ = 0.56, *P* = 0.46; interaction genotype*KCl: frequency: *F*_*1,49*_ = 0.38, *P* = 0.54, amplitude: *F*_*1,49*_ = 0.98, *P* = 0.33). (**I**) Cumulative distributions of IEI (left panels) and amplitude (right panels) of mIPSCs under 2.5 mM (upper panel) and 12.5 mM KCl condition (lower panel) showing no differences between genotypes at both conditions (2.5 mM KCl frequency: P > 0.10; amplitude; P > 0.10; wild-type n = 14 cells/4 mice; *HRas* ^*G12V/G12V*^ n = 16 cells/5 mice) (12.5 mM KCl frequency: P > 0.10, amplitude: P > 0.10; wild-type n = 10 slices/3 mice, *HRas* ^*G12V/G12V*^ n = 13 slices/4 mice). Statistical test: two-way MANOVA and K–S (for average and cumulative values, respectively).

To determine whether changes in synaptic function account for the spatial learning impairments in *HRas* ^*G12V/G12V*^ mice, we measured baseline synaptic transmission using whole-cell recordings from hippocampal CA1 pyramidal neurons. No significant differences were observed in the frequency or amplitude of spontaneous inhibitory or excitatory postsynaptic currents (sIPSCs: frequency: *t*_*44*_ = −0.39, P = 0.69; amplitude: *t*_*44*_ = 0.38, P = 0.70; sEPSCs, frequency: *t*_*44*_ = −0.32, P = 0.75; amplitude: *t*_*44*_ = 0.67, P = 0.50; Fig. 4A–D). Evoked monosynaptic IPSCs and EPSCs were also unchanged in *HRas* ^*G12V/G12V*^ mice compared to WT littermates (eEPSCs: *F*_*1,24*_ = 0.26, P = 0.61; eIPSCs: *F*_*1,19*_ = 0.01, P = 0.95; Fig. 4E–G).

The underlying mechanism for the cognitive deficits in the NF1 RASopathy mouse model has been previously shown to result from a shift in the excitatory/inhibitory balance, in which inhibitory synaptic transmission is abnormally enhanced during high frequency firing^24, 25^. Therefore, in order to investigate if a similar mechanism might be present in *HRas* ^*G12V/G12V*^ mice, we recorded spontaneous miniature inhibitory postsynaptic currents (mIPSCs) in the presence of depolarizing levels of extracellular potassium (increased from 2.5 to 12.5 mM KCl), as previously shown for *Nf1* mice^24, 25^. As expected, high extracellular potassium (12.5 mM) resulted in a significantly increased frequency of mIPSCs in both *HRas* ^*G12V/G12V*^ and WT mice (frequency: *F*_*1,49*_ = 22.28, *P* < 0.05, amplitude: *F*_*1,49*_ = 1.21, *P* = 0.28; Fig. 4H,I). However, no differences in frequency or amplitude were observed between genotypes (effect of the genotype at 2.5 mM KCl condition frequency: *F*_*1,49*_ = 1.07, *P* = 0.30, amplitude: *F*_*1,49*_ = 0.56, *P* = 0.46; interaction genotype*12.5 mM KCl frequency: *F*_*1,49*_ = 0.38, *P* = 0.54, amplitude: *F*_*1,49*_ = 0.98, *P* = 0.33; Fig. 4H,I).

**Figure 5:**
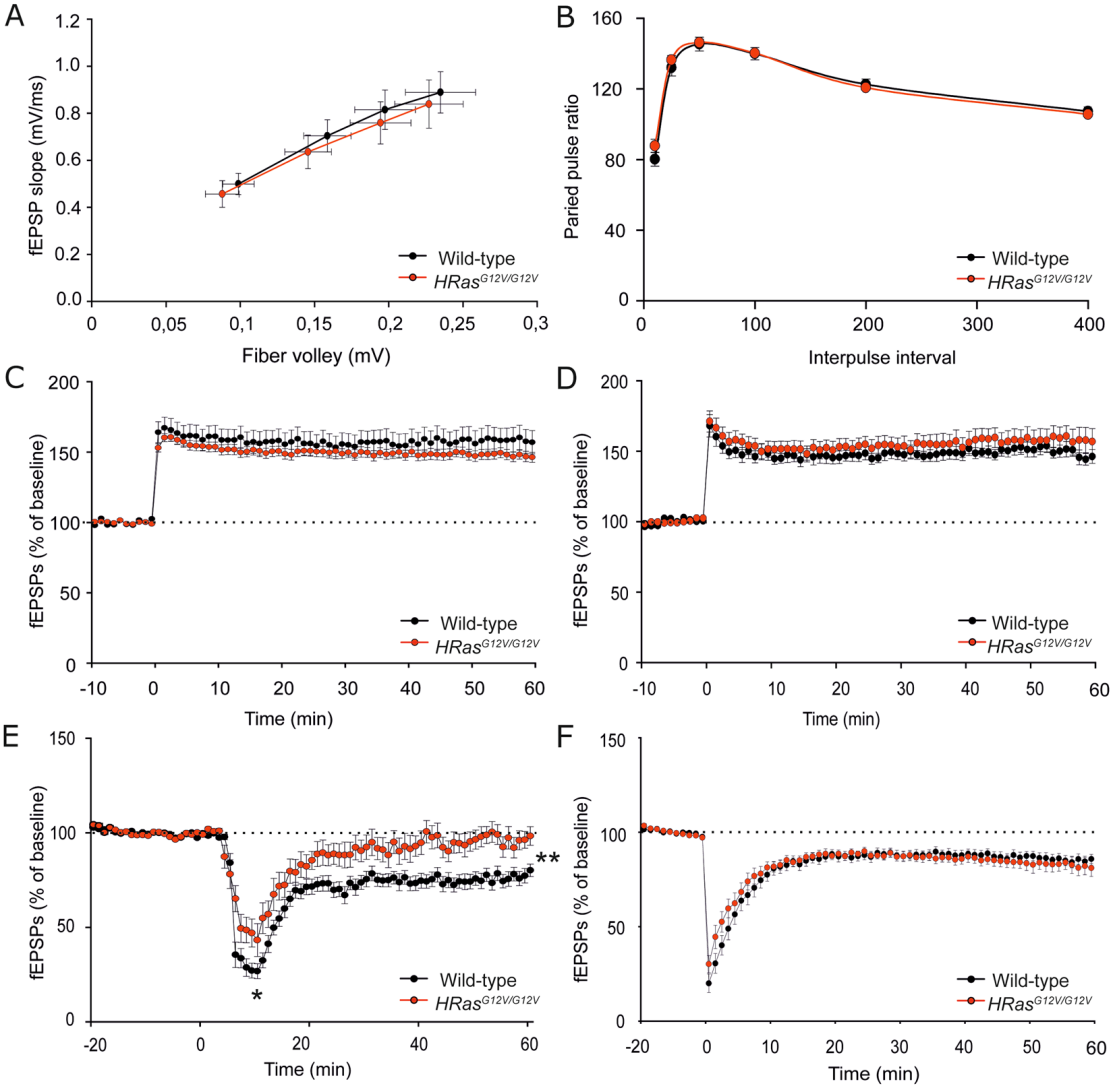
Normal long-term potentiation (LTP) but impaired mGluR-dependent long-term depression (LTD) in *HRas* ^*G12V/G12V*^ mice. (**A**) Field recordings of acute hippocampal slices revealed normal basal synaptic transmission in *HRas* ^*G12V/G12V*^ mice (effect of genotype: fiber volley: *F*_*1,53*_ = 0.15, *P* = 0.70, fEPSP slope: *F*_*1,53*_ = 0.17, *p* = 0.68). Data are presented as mean ± SEM from wild-type n = 29 slices/4 mice and *HRas* ^*G12V/G12V*^ n = 26 slices/4 mice. (**B**) Paired-pulse ratio is not altered in *HRas* ^*G12V/G12V*^ mice compared to WT controls (*F*_*1,14*_ = 0.36, *P* = 0.56). Data are presented as mean ± SEM from: for 2.5 mM KCl: *HRas* ^*WT/WT*^ n = 25 slices/6 mice, *HRas* ^*G12V/G12V*^ n = 26 slices/8 mice. (C-D) Normal LTP at Schaffer collateral-CA1 synapses in *HRas* ^*G12V/G12V*^ induced by (**C**) theta burst stimulation (TBS, effect of genotype: *F*_*1,20*_ = 0.92, *P* = 0.51) or by (**D**) 100 Hz stimulation. Data are presented as mean ± SEM from for (**C**) wild-type n = 9 slices/4 mice, *HRas* ^*G12V/G12V*^ n = 13 slices/4 mice n = 29 slices/4mice and for (**D**) wild-type n = 9 slices/4 mice, *HRas* ^*G12V/G12V*^ n = 7 slices/4 mice. (**E**) DHPG application (50 μM, 5 min) induced stable long-term depression (LTD) in WT mice. Impaired LTD was observed in *HRas* ^*G12V/G12V*^ mice (*F*_*1,34*_ = 13.29, *P* < 0.01). Data are presented as mean ± SEM from wild-type n = 20 slices/3 mice, *HRas* ^*G12V/G12V*^ n = 16 slices/3 mice. (**F**) NMDA receptor-dependent LTD, induced by low frequency stimulation (LFS), was indistinguishable between wild-type and *HRas* ^*G12V/G12V*^ mice (*F*_*1,26*_ = 0.40, *P* = 0.53). Data are presented as mean ± SEM from wild-type n = 17 slices/4 mice, *HRas* ^*G12V/G12V*^ n = 11 slices/3 mice. Statistical test for (**A–F)**: one-way repeated measures ANOVA. Data are expressed as percentage of the baseline prior to the stimulation protocol.

To further examine whether the *HRas* ^*G12V/G12V*^ mutation and the resulting increase of pERK signaling might affect short- or long-term forms of synaptic plasticity, we recorded field excitatory postsynaptic potentials (fEPSPs) from hippocampal Schaffer collateral (SC)-CA1 synapses. Consistent with the whole-cell data, baseline synaptic transmission (defined by the relationship between the postsynaptic fEPSP slope and presynaptic fiber volley amplitude) was similar between *HRas* ^*G12V/G12V*^ mice and their WT littermates (fiber volley: *F*_*1,53*_ = 0.15, *P* = 0.70, fEPSP slope: *F*_*1,53*_ = 17, *P* = 0.68; Fig. 5A). Furthermore, paired-pulse facilitation (PPF), which represents a presynaptically-mediated short-term form of plasticity, was also normal in *HRas* ^*G12V/G12V*^ mice (*F*_*1,14*_ = 0.36, *P* = 0.56, Fig. 5B). Since long-term plasticity (LTP) induced by theta-burst stimulation (TBS) is impaired in *Nf1* ^8, 25^, *Ptpn11* ^10^, *Spred1* mice^11^ and conditional *Kras* ^*G12V*^ mice^26^ (see Table 1), we tested LTP induced by a theta-burst stimulation protocol and a high frequency (100 Hz) stimulation protocol. Notably, both TBS-induced LTP and 100Hz-induced was similar between genotypes (TBS: *F*_*1,20*_ = 0.92, *P* = 0.51; 100 Hz: *F*_*1,14*_ = 0.80, *P* = 0.61; Fig. 5C,D). Taken together, these findings suggest that although constitutively active HRAS impairs spatial learning, it does not affect hippocampal synaptic transmission nor short- or long-term synaptic potentiation.

### Impaired mGluR-LTD in *HRas*^*G12V/G12V*^ mice

Metabotropic glutamate receptor-dependent long-term depression (mGluR-LTD), a form of synaptic plasticity resulting in decreased synaptic efficacy, has been shown to strongly depend on activity of the RAS-ERK pathway^27, 28^. Therefore, we hypothesized that hippocampal mGluR-dependent LTD might be affected in *HRas* ^*G12V/G12V*^ mice. Hippocampal slices from *HRas* ^*G12V/G12V*^ mice and their wild-type littermates were treated with DHPG (50 μM, 5 min), a specific agonist of group I (mGluR1 and mGlur5) metabotropic glutamate receptors, to induce LTD. This resulted in a substantial initial depression of synaptic transmission in WT mice and significantly less pronounced depression in *HRas* ^*G12V/G12V*^ mice (*F*_*1,34*_ = 6.72, *P* < 0.05; Fig. 5E). This depression was followed by a robust long-lasting depression in fEPSCs amplitude in WT mice whereas it quickly returned to near baseline levels in *HRas* ^*G12V/G12V*^ mice, indicating that mGluR-dependent LTD cannot be stably expressed in *HRas* ^*G12V/G12V*^ mice (*F*_*1,34*_ = 13.29, *P* < 0.01; Fig. 5E). In order to determine the pathway and receptor specificity of the LTD deficit in *HRas* ^*G12V/G12V*^ mice, N-methyl D-aspartate receptor-dependent LTD (NMDAR-LTD) was induced using low-frequency stimulation (LFS). Notably, LFS-induced LTD was similar between *HRas* ^*G12V/G12V*^ mice and WT littermates (*F*_*1,26*_ = 0.40, *P* = 0.53; Fig. 5F), indicating that HRAS^G12V^ specifically affects mGluR-dependent LTD.

### Lovastatin does not rescue the spatial learning deficit in *HRas*^*G12V/G12V*^ mice

**Figure 6:**
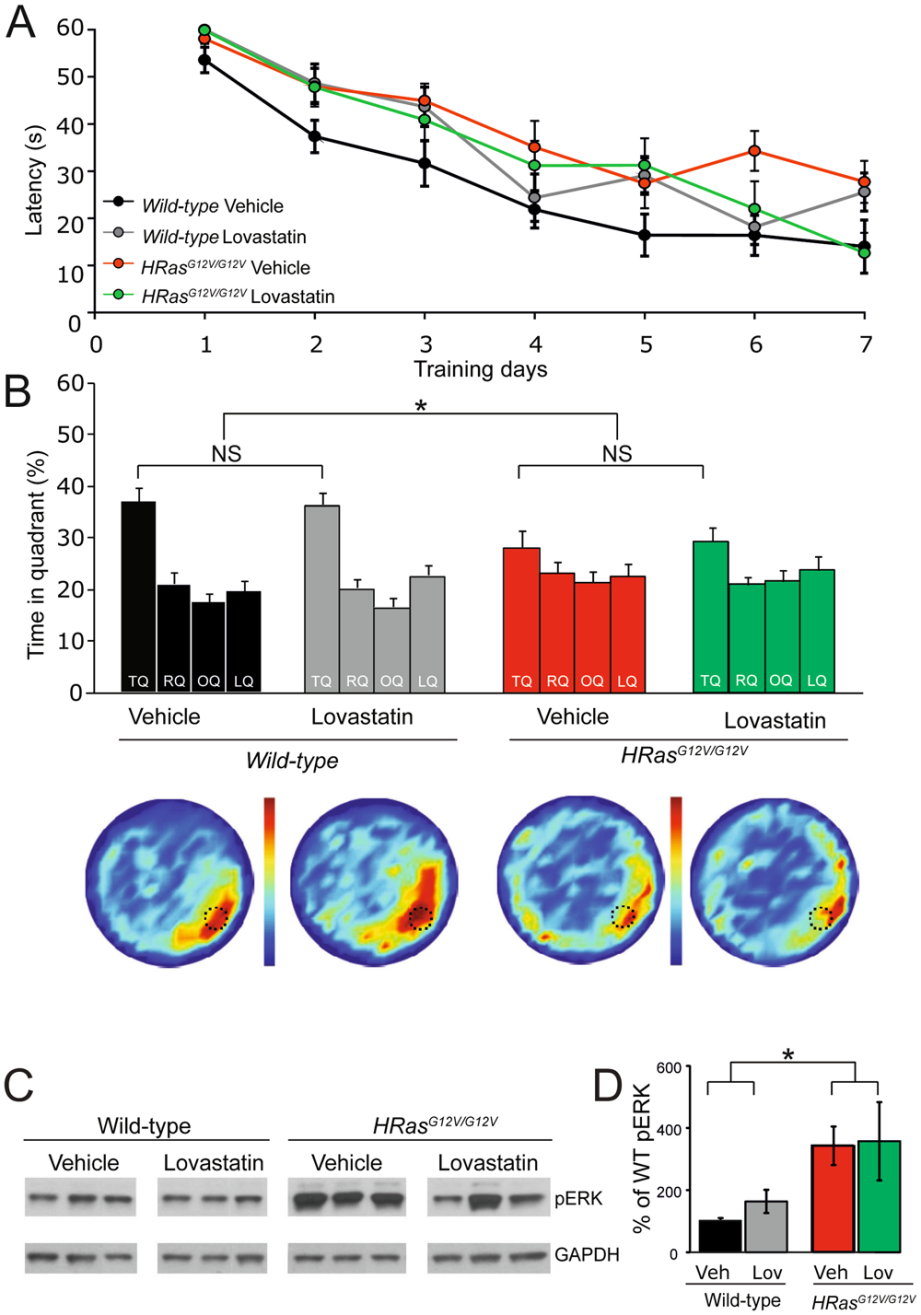
Lovastatin does not rescue learning deficits or increased pERK level in *HRas* ^*G12V/G12V*^ mice. (**A**) Average latency to platform for each day of training, per genotype. A Significant effect of learning on the latencies of all groups was observed (within subject comparison: effect of time: *F*_*1,58*_ = 46.80, P < 0.01). On average, *HRas* ^*G12V/G12V*^ mice (both groups: lovastatin and vehicle treated) showed significantly longer latencies compared to wild-type littermates (between subjects comparison: effect of genotype: *F*_*1,58*_ = 5.89, P < 0.05). No effect of treatment on latencies was observed (between subjects comparison: effect of treatment: *F*_*1,58*_ = 0.23, P = 0.63). (**B**) Probe trial data of watermaze learning shows that lovastatin did not rescue the learning deficit in *HRas* ^*G12V/G12V*^ mice (effect of treatment: P = 0.76; genotype*treatment interaction P = 0.79). TQ = target quadrant, RQ = adjacent right quadrant, LQ = adjacent left quadrant, OQ = opposite quadrant. Heat plots represent all search tracks combined, in which the color indicates the mean time spent at that position in the pool. Data are presented as mean ± SEM. *P < 0.05. NS: Not significant. Number of mice: vehicle group wild-type n = 17; *HRas* ^*G12V/G12V*^ n = 12; treated group: wild-type n = 17; *HRas* ^*G12V/G12V*^ n = 14. (**C**) Western blots of hippocampal lysates of the WT and *HRas* ^*G12V/G12V*^ mice showing an increased pERK level in untreated *HRas* ^*G12V/G12V*^ mice, but no effect of lovastatin on pERK level. (**D**) Quantification of Western blots. A significant effect of genotype was found in pERK level (*F*_*1,20*_ = 6.48, P < 0.05). No main effect of treatment on pERK could be detected (*F*_*1,20*_ = 0.21, P = 0.65; interaction genotype*treatment: *F*_*1,20*_ = 0.08, P = 0.78). Data are presented as mean ± SEM normalized to vehicle group wild-type. n = 5; *HRas* ^*G12V/G12V*^ n = 5; treated group: wild-type n = 7; *HRas* ^*G12V/G12V*^ n = 7. Three mice per group are depicted in the example blot. Statistical test: two-way MANOVA.

As shown in Fig. 1, a major biochemical alteration exhibited in *HRas* ^*G12V/G12V*^ mice is hyper activation of the RAS-ERK pathway, resulting from the constitutive activation of HRAS. The G12V mutation reduces the intrinsic GTPase activity of HRAS protein and thereby prolongs its GTP-bound state^29, 30^. Considering the fact that transition of GTD- to GTP-bound RAS relies on proper anchoring to the plasma membrane by means of a lipid (farnesyl)-based plasma-membrane-targeting motif, interfering with its membrane recruitment is an established strategy to reduce RAS activity. Notably, treatment with lovastatin, a specific inhibitor of the rate-limiting enzyme in cholesterol biosynthesis (HMG-CoA reductase), can reduce RAS farnesylation and activity, and has been previously shown to reverse spatial learning deficits in the *Nf1* ^31^ and *Ptpn11* ^10^ mouse models for RASopathies. Therefore, we tested whether the same lovastatin treatment would also rescue the spatial learning deficit of *HRas* ^*G12V/G12V*^ mice. Mice were treated with lovastatin (10 mg/kg) once daily for 3 days prior to and during hidden platform Morris water maze training (daily injection 6 h prior to the training sessions). Although latencies to find the platform significantly decreased in all groups during training (within-subjects comparison, effect of time: *F*_*1,58*_ = 46.80, P < 0.01), *HRas* ^*G12V/G12V*^ mice showed significantly longer latencies compared to wild-type littermates (between-subjects comparison, effect of genotype: *F*_*1,58*_ = 5.89, P < 0.05, Fig. 6A). Moreover, there was no effect of Lovastatin treatment (genotype*treatment interaction: *F*_*1,58*_ = 0.23, P = 0.61). The effect of lovastatin on spatial learning in both groups was assessed in a probe trial given after the 7^th^ day of the training. As shown in Fig. 6B, wild-type mice treated with vehicle spent significantly more time searching in the target quadrant (TQ) than *HRas* ^*G12V/G12V*^ mice, confirming the impairment in spatial learning (TQ of *HRas* ^*WT/WT*^ vehicle vs. TQ of *HRas* ^*G12V/G12*^ vehicle *t*_*27*_ = 2.18, P < 0.05). However, lovastatin did not improve spatial learning of either group (effect of treatment: *F*_*1,58*_ = 0.09, P = 0.76; genotype*treatment interaction: *F*_*1,58*_ = 0.07, P = 0.79; Fig. 6B).

To further characterise the effect of lovastatin treatment, we quantified pERK(42/44) in hippocampal lysates of WT and in *HRas* ^*G12V/G12V*^ mice following the completion of behavioural testing. As described above, pERK1/2 level was significantly higher in *HRas* ^*G12V/G12V*^ mice compared to wild-type littermates (effect of the genotype: *F*_*1,10*_ = 6.48, P < 0.05), but unaffected by lovastatin treatment (effect of the treatment: *F*_*1,10*_ = 0.21, P = 0.65; interaction genotype*treatment: *F*_*1,20*_ = 0.08, P = 0.78; Fig. 6C,D). Taken together, these data suggest that treatment with lovastatin using the same dosing regimen that was sufficient to rescue special learning and pERK hyper-activation in the *Nf1* ^31^ and *Ptpn11* ^10^ RASopathy models, is not effective in rescuing the spatial learning deficit of *HRas* ^*G12V/G12V*^ mice.

### Acute inhibition of MEK rescues deficit in mGluR-LTD in *HRas*^*G12V/G12V*^ mice

**Figure 7:**
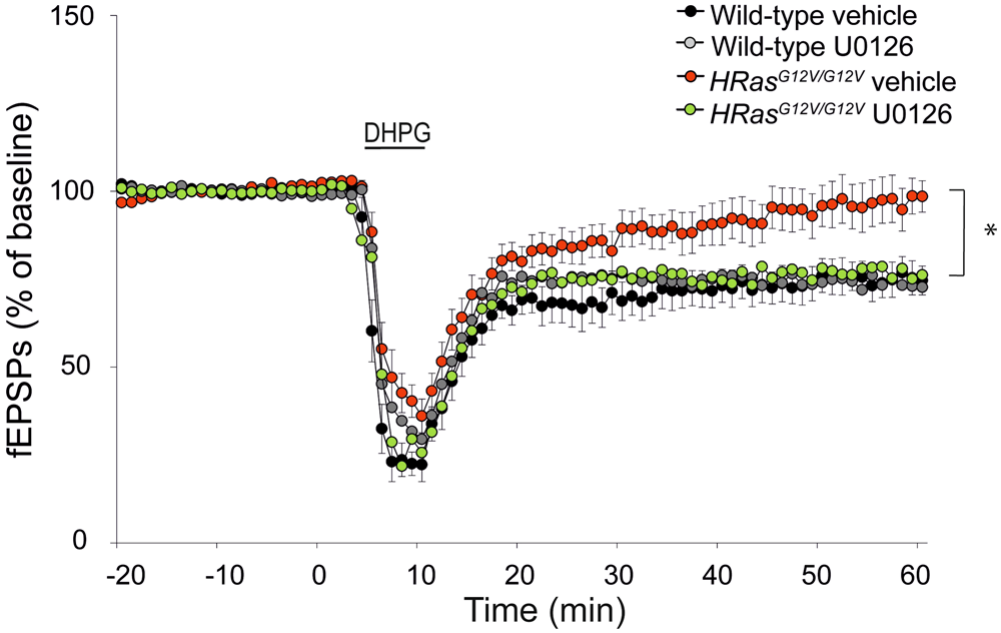
Acute inhibition of MEK rescues deficit in mGluR-LTD in *HRas* ^*G12V/G12V*^ mice. Induction of chemical LTD by application of DHPG for 5 min (50 μM), induced stable depression of synaptic efficacy in slices of WT and only temporarily depression in *HRas* ^*G12V/G12V*^ slices, both treated with vehicle solution (effect of genotype, vehicle treated group: *F*_*3,63*_ = 8.53, P < 0.01). Pre-incubation of slices with a MEK-inhibitor, U0126, did not affect wild-type LTD (wild-type vehicle vs. wild-type treated, P > 0.05), but rescued the LTD-deficit in *HRas* ^*G12V/G12V*^ (wild-type treated vs. *HRas* ^*G12V/G12V*^ treated, p < 0.05). Data are presented as mean ± SEM from vehicle treated wild-type. Vehicle treated: wild-type n = 17 slices/5 mice, *HRas* ^*G12V/G12V*^ n = 17 slices/5 mice and drug treated: wild-type n = 16 slices/4 mice, *HRas* ^*G12V/G12V*^ n = 20 slices/5 mice. Statistical test: repeated measure ANOVA and *post-hoc* Bonferroni correction.

Previous studies have demonstrated an important role of the RAS-ERK pathway in mediating the expression of mGluR-LTD^27, 32^. Hence we hypothesized that the mGluR-LTD deficit exhibited by *HRas* ^*G12V/G12V*^ mice might be caused by hyperactive ERK. If this model is valid, the impaired mGluR-LTD in *HRas* ^*G12V/G12V*^ slices should be able to be rescued by a MEK inhibitor, which would decrease ERK activation. Therefore, we induced mGluR-LTD by administering hippocampal slices with DHPG in addition to pre-treatment with either vehicle or a subthreshold dose of a selective and membrane-permeable MEK-inhibitor (U0126, 1 μM) which was previously shown not to affect WT mGluR-LTD at this concentration^28^. Application of DHPG induced stable depression of synaptic efficacy in wild-type slices, but only a brief depression in *HRas* ^*G12V/G12V*^ slices treated with vehicle solution (effect of genotype, vehicle treated group: *F*_*3,63*_ = 8.53 P < 0.01; Fig. 7), thereby replicating the mGluR-LTD deficit in *HRas* ^*G12V/G12V*^ mice. Notably, pre-incubation of slices with the MEK-inhibitor U0126 rescued the LTD deficit in *HRas* ^*G12V/G12V*^ (WT vehicle vs*. HRas* ^*G12V/G12V*^ treated, Bonferroni’s post-hoc test P < 0.05) without any discernible effect on LTD in WT littermates (WT vehicle vs. WT treated, Bonferroni’s post-hoc test P > 0.05). Together, these results indicate that the deficit in mGluR-LTD observed in *HRas* ^*G12V/G12V*^ mice is indeed caused by hyperactive RAS-ERK signaling, and that acute inhibition of this pathway with a MEK inhibitor restores normal induction and expression of mGluR-LTD in *HRas* ^*G12V/G12V*^ mice.

### Restoring pERK levels in *HRas*^*G12V/G12V*^ mice by a MEK inhibitor does not rescue the spatial learning deficit or increased brain weight

**Figure 8:**
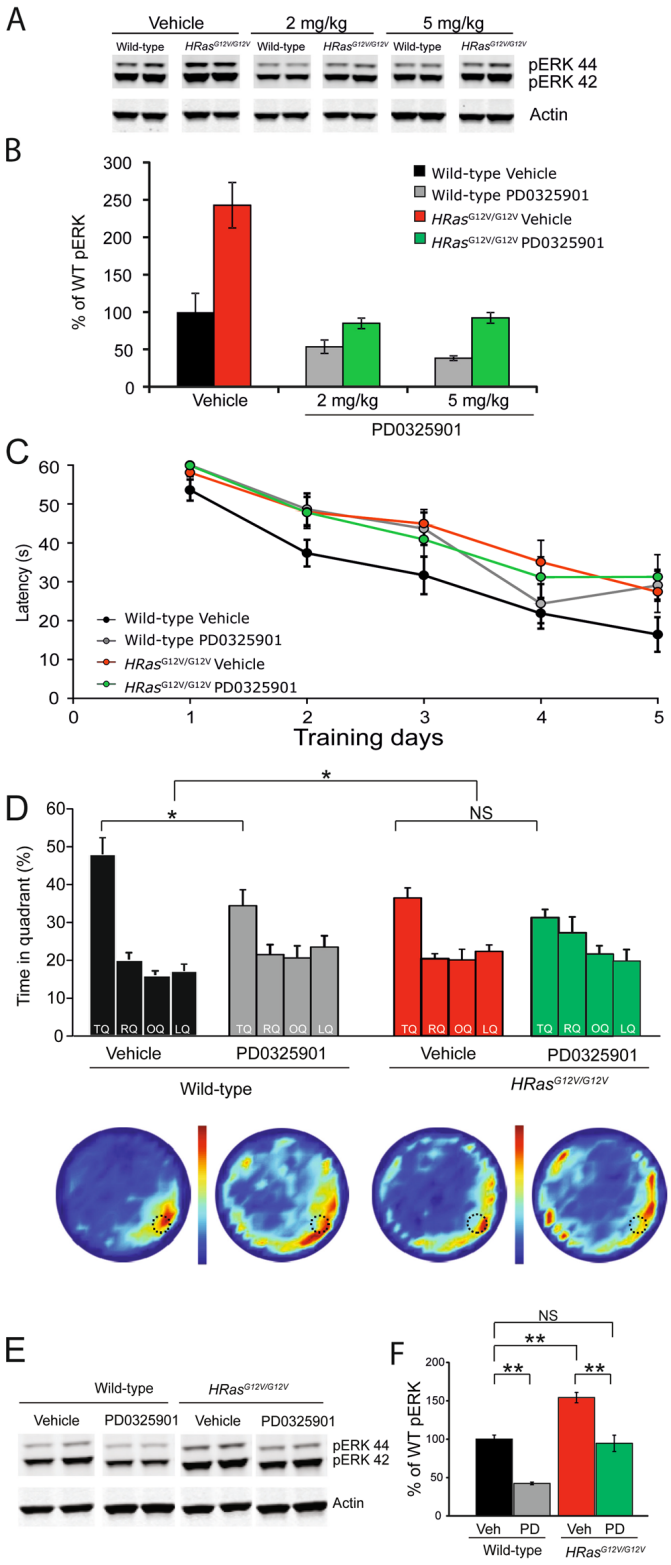
Inhibition of MEK with PD0325901 restores pERK levels but does not rescue the spatial learning deficit. (**A**) Western blots of hippocampal lysates of WT and *HRas* ^*G12V/G12V*^ mice treated with vehicle, 2 and 5 mg/kg of PD0325901. (**B**) Quantification of Western blots showing that both doses used affect pERK levels in both wild-type and *HRas* ^*G12V/G12V*^ mice. For both 2 and 5 mg/kg: effect of genotype: P < 0.01, effect of treatment: P < 0.01, interaction genotype*treatment P < 0.01. Data are presented as mean ± SEM from 6 mice per group. Two mice per group are depicted in the example blot. Statistical test: two-way ANOVA. (**C**) Average latency to platform for each day of training, per genotype. On average longer latencies were observed in vehicle and PD0325901 drug treated *HRas* ^*G12V/G12V*^ mice (effect of genotype P < 0.01). (**D**) Spatial memory assessed in a probe trial reveals a significant effect of genotype (P < 0.05) on time spent in the target quadrant (TQ). Although PD0325901 significantly reduced performance of wild-type mice, it showed no effect on mutant mice and no interaction of genotype and treatment was observed (P = 0.28). TQ = target quadrant, RQ = adjacent right quadrant, LQ = adjacent left quadrant, OQ = opposite quadrant. Heat plots present the data of all tracks combined, in which the color indicates the mean time spent at that position in the pool. Data are presented as mean ± SEM from: vehicle group: wild-type n = 14 mice; *HRas* ^*G12V/G12V*^ n = 14 mice; treated group: wild-type n = 13 mice; *HRas* ^*G12V/G12V*^ n = 13 mice. Statistical test: repeated measures two-way ANOVA and two-way MANOVA, respectively. (**E**) Western blot of hippocampal tissue taken from WT and *HRas* ^*G12V/G12V*^ mice after water maze training and concurrent treatment with either vehicle or PD0325901. (**F**) Quantification of Western blots. A significant effect of genotype (*F*_*1,37*_ = 47.83 P < 0.01) and treatment (P < 0.01) on pERK was observed. No interaction effect of genotype and treatment was found (P = 0.37). Data are presented as mean ± SEM from n = 10 mice per group. Statistical test: unpaired two-tailed *t*-test.

Given that the mGluR-LTD deficit of *HRas* ^*G12V/G12V*^ slices can be rescued by a MEK inhibitor, we investigated whether the learning phenotype observed in *HRas* ^*G12V/G12V*^ could be reversed by treatment with a MEK inhibitor. For these experiments, we used the allosteric MEK inhibitor PD0325901 which has good bioavailability and is often used for *in vivo* studies, including studies targeting the brain^9, 33–35^. Although previous studies have shown a rescue of several phenotypes in an adult mouse model of Noonan syndrome using PD0325901 at 5 mg/kg bodyweight^35^, we sought to identify a lower effective dose, since inhibition of the RAS-ERK pathway results in learning deficits^36^. Mice were injected daily for 7 days with 2 or 5 mg/kg of PD0325901 and sacrificed 2 h after their final scheduled injection to assess pERK levels. As shown in Fig. 8A,B, pERK was significantly higher in vehicle-treated *HRas* ^*G12V/G12V*^ mice compared to vehicle treated WT littermates. Treatment with the MEK-inhibitor showed a dose-dependent effect on pERK in both *HRas* ^*G12V/G12V*^ mice as well as wild-type littermates (effect of genotype: *F*_*1,34*_ = 6.88, P < 0.05). At only 2 mg/kg of PD0325901, a significant effect of treatment was already observed (*F*_*1,34*_ = 38.95, P < 0.01), in which pERK levels in *HRas* ^*G12V/G12V*^ mice were similar to vehicle-treated wild-type littermates. Hence, we chose to proceed with the 2 mg/kg daily dose, beginning 3 days prior to water maze training. All groups of mice showed a significant reduction in the latency to find the platform during training (effect of time: *F*_*1,55*_ = 49.50, P < 0.01). However, *HRas* ^*G12V/G12V*^ mice exhibited significantly longer search latencies, regardless of treatment (effect of genotype: *F*_*1,55*_ = 8.97, P < 0.01; Fig. 8C).

Spatial learning of wild-type and *HRas* ^*G12V/G12V*^ littermates, treated either with vehicle or PD0325901, was assessed in a probe trial after 5 days of training, a time point in which the wild-type mice showed already a strong preference for the target quadrant. Although it was notable that under these conditions vehicle treated *HRas* ^*G12V/G12V*^ mice also showed a significant preference for the target quadrant, we again observed a significant effect of genotype with respect to time spent in the target quadrant, replicating the spatial learning deficit in *HRas* ^*G12V/G12V*^ mice (effect of genotype: *F*_*1,51*_ = 6.07, P < 0.05; Fig. 8D). In addittion, a significant effect of treatment was observed this time (*F*_*1,51*_ = 7.41 P < 0.05; Fig. 7D), but this was mediated by a decrease in performance of the treated mice. There was no significant interaction between genotype and treatment (*F*_*1,51*_ = 1.21, P = 0.28).

In order to confirm that daily PD0325901 treatment reduced pERK levels in *HRas* ^*G12V/G12V*^ mice to that of their wild-type littermates, we performed Western blot analysis of hippocampal lysates taken from WT and *HRas* ^*G12V/G12V*^ mice immediately following the conclusion of the probe trial. Quantification of Western blots revealed a significantly higher level of pERK in vehicle-treated *HRas* ^*G12V/G12V*^ mice compared to vehicle treated wild-type mice (effect of genotype *F*_*1,37*_ = 47.83, P < 0.01) with a significant decrease in pERK after PD0325901 treatment in both WT and *HRas* ^*G12V/G12V*^ mice (effect of treatment *F*_*1,37*_ = 35.49, P < 0.01; genotype*treatment *F*_*1,37*_ = 0.81, P = 0.38; Fig. 8E,F). Importantly, pERK levels in PD0325901-treated *HRas* ^*G12V/G12V*^ mice were similar to their vehicle-treated WT littermates, and thus reduced to normal levels.

**Figure 9:**
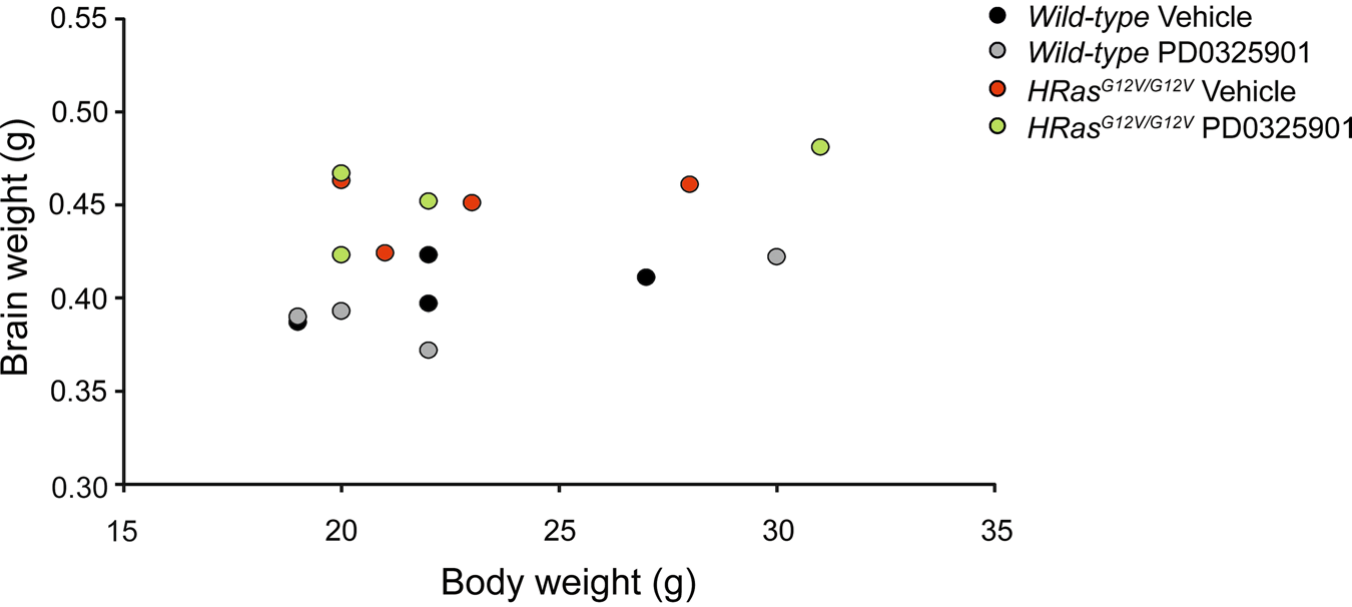
Treatment with MEK inhibitor does not reduce the increased brain weight of *HRas* ^*G12V/G12V*^ mice. (**A**) Daily treatment with 2 mg/kg of PD0325901 for 4 weeks does not reduce the increased brain weight observed in *HRas* ^*G12V/G12V*^ mice (effect of genotype: brain weight: *F*_*1,12*_ = 28.20, P < 0.01; body weight: *F*_*1,12*_ = 0.05, P = 0.83; effect of treatment*:* brain weight*: F*_*1,12*_ = 0.45, P = 0.84, body weight: *F*_*1,12*_ = 0.01, P = 0.91; interaction genotype*treatment: brain weight: *F*_*1,12*_ = 0.65, P = 0.43; body weight: *F*_*1,12*_ = 0.00, P = 1.00). Data are presented as individual values of brain vs. body weight from n = 4 mice per each group. Statistical test: two-way MANOVA.

We also examined whether PD0325901 treatment influenced the brain weight of WT and *HRas* ^*G12V/G12V*^ mice. *HRas* ^*G12V/G12V*^ mice again exhibited significantly heavier brains, independent of body weight (effect of genotype: brain weight: *F*_*1,12*_ = 28.20, P < 0.01; body weight: *F*_*1,12*_ = 0.05, P = 0.83). However, we observed no effect of PD0325901 treatment (effect of genotype: brain weight*: F*_*1,12*_ = 0.45, P = 0.84, body weight: *F*_*1,12*_ = 0.01, P = 0.91; interaction genotype*treatment: brain weight: *F*_*1,12*_ = 0.65, P = 0.43; body weight: *F*_*1,12*_ = 0.00, P = 1.00; Fig. 9). Taken together, these data suggest that although acute MEK inhibition with PD0325901 successfully restores the level of pERK in adult *HRas* ^*G12V/G12V*^ mice to that of WT mice, the MEK inhibitor does not rescue the morphological alterations or spatial learning deficits under these conditions.

## Discussion

Here we report that a mouse model for Costello syndrome (CS), which carries an activating mutation in the *HRAS* gene, shows hyperactivation of the RAS-ERK pathway, learning deficits, structural brain abnormalities, and alterations in synaptic functioning. Although we studied homozygous *HRas* ^*G12V/G12V*^ mice, these mice express the HRAS^G12V^ protein at approximately 50%, and hence their levels of oncogenic HRAS protein resemble that of heterozygous CS patients. The reduced HRAS protein expression is possibly caused by the IRES-β-geo cassette inserted into the 3′UTR of the *HRas* ^*G12V*^ targeting vector, as reduced expression is not observed in an independent line of *HRas* ^*G12V*^ mice^37^.

Previously it has been shown that pERK was not increased in this mouse line^16, 17^, whereas we consistently observed a highly significant increase in pERK levels in hippocampal lysates (Fig. 1). This could be due to brain-region specific variability of ERK expression and activation levels resulting in no significant difference observed in total brain lysates^38^. Indeed, immunolabeling as shown by Viosca *et al*. clearly indicate increased pERK staining in CA3 mossy fiber region of hippocampus suggesting that, at least, locally pERK levels are enhanced.

We have deliberately focused our study on CS, as the RAS protein is the central protein of the RAS-ERK signaling pathway. In contrast to germline *KRAS* ^*G12V*^ mutants, the germline *HRas* ^*G12V*^ mutation is not lethal, which allowed us to directly study the molecular, cellular, and behavioral consequences of a constitutively active HRAS protein present from germline. Furthermore, it also enabled us to determine if there were differences in phenotype- or treatment efficacy compared to other mouse models of RASopathies caused by mutations in up-stream regulators of HRAS protein, such as NF1, SHP2/PTPN11, and SPRED1.

In accordance with several previous studies using adult transgenic *HRas* ^*G12V*^ overexpressing mice, we observed brain and neuronal hypertrophy in *HRas* ^*G12V/G12V*^ mutant mice^20, 21^. Moreover, an enlarged corpus callosum was observed after bi-allelic inactivation of *Nf1* ^9^. These morphological changes could not be rescued by acute treatment with the MEK-inhibitor PD0325901 during adulthood. A recent study reported that aberrant RAS signaling in the developing brain (E14-P7) resulted in an increased production of cortical neurons and morphological deficits^39^. In addition, other studies have demonstrated a clear requirement for intact ERK activity in controlling cortical development, including fibroblast growth factor (FGF)-stimulated cortical progenitor differentiation and neurogenesis^2, 40^. Moreover, a recent study showed increased proteoglycan expression in astrocytes derived from iPSCs from CS patients as well as in astrocytes of mutant *HRas* ^*G12S*^ mice^41^. These studies highlight the importance of proper control of the RAS-ERK signaling during brain development, and suggest that adult treatment could be too late to restore the behavioural phenotype.

As summarized in Table 1, mouse models for Costello Syndrome (CS), Neurofibromatosis I (NF1), Noonan Syndrome (NS), and Legius Syndrome show similar phenotypes including hyperactivity of the RAS-ERK pathway, learning deficits in the water maze, and impaired synaptic plasticity, which confirm the widespread observation of overlapping disease manifestations across patients with different RASopathies. Given the shared biochemical alteration of increased hyperactivity of the RAS-ERK pathway, and the indisputable role of RAS-ERK signaling in LTP and memory formation^36, 42^, it seemed reasonable to assume that the plasticity deficits observed in these RASopathy mouse models might all be caused by the same mechanism. However, a closer look at the specific cellular mechanisms reveals substantial differences between the RASopathy mouse models. For instance, neither the cell-type specific increase of inhibition found in *Nf1* mutants^24^ and a conditional *Kras* ^*G12V*^ mutant^26^, nor the increase of glutamatergic synaptic transmission observed in the NS model^10^ were observed in *HRas* ^*G12V/G12V*^ mice. In addition, we and others showed that LTP induced by theta-burst stimulation is impaired in all RASopathy mouse models published so far: *Nf1* ^8, 25^, *Ptpn11* ^10^, *Spred1* mice^11^ and conditional *Kras* ^*G12V*^ mice^26^. However, despite the strong ERK activation, LTP was unaffected in *HRas* ^*G12V/G12V*^ mice. The phenotypic differences between the mutants could be caused by differences in RAS-ERK activation (Table 1), by differences in cell-type specific expression, and/or by differential regulation during development. These possibilities could also explain increased LTP in transgenic mice expressing the same HRAS protein (HRAS^G12V^) driven by the *CAMK2* promoter^43^ resulting in transgenic expression restricted to glutamatergic pyramidal neurons in the cortex and hippocampus. Moreover, there could be subtle differences in the intracellular location of HRAS, NF1, SPRED1, PTPN11 and KRAS which diverge in their relative subcellular signaling mechanisms^44, 45^. Lastly, some of these proteins may have RAS-independent functions. For example, it was recently reported that NF1 interacts with hyperpolarization-activated cyclic nucleotide-gated (HCN) channels for which mutations in *Nf1* cause attenuations of *I*_h_ current selectively in GABAergic interneurons^25^. Treatment with the HCN channel agonist lamotrigine during adulthood rescued the cognitive deficits in two different *Nf1* mouse models, thereby establishing an important function of HCN channels in mediating the NF1 phenotype. In contrast, HCN function is unaffected in *HRas* ^*G12V/G12V*^ mice^25^.

Previous studies have shown that mGluR-dependent LTD is sensitive to changes in ERK activity. High concentrations of the MEK-inhibitor U0126 (20 µM) impair mGluR-LTD^27, 28^, while a lower dose of U0126 (1 µM), which did not affect mGluR-LTD in WT mice, was sufficient to rescue the mGluR-LTD deficit in *HRas* ^*G12V/G12V*^ mice. This counter-intuitive result suggests that mGluR-LTD is sensitive to both hypo- and hyper activation of RAS-ERK pathway. Possibly, due to the high levels of RAS-ERK signaling in our mouse model, mGluR-mediated LTD was inhibited, and pre-treatment with a low dose of U0126 brought ERK signaling back into the normal range. To determine the mechanism underlying the impairment of mGluR-LTD in *HRas* ^*G12V/G12V*^ mice will require further investigation. In addition, it will be important to assess whether mGluR-LTD is altered the NF1, Legius Syndrome, or Noonan syndrome RASopathy mouse models.

Until recently, many symptoms of neurodevelopmental disorders, including RASopathies and mTOR-pathies^3^, were thought to be untreatable in adulthood due to their neurodevelopmental alterations, including gross brain dysmorphology and/or neuronal wiring/connectivity. Remarkably, candidate therapies implemented in NF1 and Noonan syndrome mouse models have shown extensive phenotypic rescue, including behavioral, synaptic, and molecular alterations. For instance, spatial learning deficits in adult *Nf1* ^+/−^ mice were rescued by treatment with farnesyl-transferase inhibitor (FTI), which reduces RAS activity by interfering with the farnesylation of RAS, a post-translational modification that allows RAS to be targeted to the membrane^8, 46^. Moreover, a behavioral rescue was shown after treatment of adult *Nf1* ^+/−^ and *Ptpn11* ^*D61G/*+^ mice for 3–4 weeks with lovastatin, which affects the synthesis of farnesyl^10, 31^. Furthermore, lovastatin reduced pERK levels in *Nf1* ^+/−^ and *Ptpn11* ^*D61G/*+^ mice, and reduced pERK-dependent protein synthesis in the mouse model of Fragile X Syndrome, indicating its potency in restoring alterations of the RAS-ERK pathway^10, 31, 47^. Unfortunately however, we found that implementation of the identical lovastatin treatment regimen was unable rescue learning deficits and pERK levels in adult *HRas* ^*G12V/G12V*^ mice (Fig. 6). It is likely that the G12V mutation, leads to much higher activation of the RAS-ERK pathway than mutations in upstream regulators of RAS protein, such as NF1 or PTPN11/SHP2 and therefore might be less responsive to lovastatin. In support of this, extensive studies of the effects of lovastatin as a potential cancer therapy have revealed that the concentration of statins required to significantly reduce farnesylation of RAS proteins with oncogenic mutations such as G12V are impossible to achieve *in vivo* without substantial side effects^48, 49^.

A recent study has shown that the MEK inhibitor SL327 restored learning, LTP, and ERK signaling in adult mouse models for Noonan Syndrome, caused by mutations in *PTPN11* ^10^ (Table 1). Surprisingly, even though we observed that the mGluR-LTD deficit in *HRas* ^*G12V/G12V*^ mice can be rescued by a lower concentration of MEK inhibitor, treatment of *HRas* ^*G12V/G12V*^ mice with 2 mg/kg of PD0325901 (a MEK-inhibitor which has good bioavailability and is currently in clinical trials)^9, 33–35^ successfully restored pERK levels but was insufficient to rescue the spatial learning impairment. Recently, it was also shown that treating adult conditional *KRas* ^*G12V*^ mice with PD0325901 failed to rescue the behavioural phenotype^26^. There are several possible explanations for our failure to rescue the behavioural phenotype of *HRas* ^*G12V/G12V*^ mice. First, although we chose a dose that resulted in a normalization of pERK levels, it may be possible that we have not used an optimal dose. Second, it might be that the observed morphological brain abnormalities interfere with normal learning, despite acutely normalized levels of RAS-ERK signaling. Lastly, it could be that successful rescue of the learning deficit of *HRas* ^*G12V/G12V*^ mice with lovastatin or MEK inhibitors requires treatment earlier in neurodevelopment. We recently demonstrated that adult gene-reactivation in a mouse model for Angelman Syndrome rescued LTP but not the cognitive and motor impairments, while early developmental gene reactivation led to a full rescue of all phenotypes, thereby suggesting a therapeutic critical period for achieving effective treatment in certain neurodevelopmental disorders^50^.

Our findings in the *HRas* ^*G12V/G12V*^ mouse model, as well as emerging findings in the NF1 and Noonan syndrome mouse models^10, 25^, suggest that the mechanisms underlying learning deficits in RASopathies can be highly distinct, for which a universal treatment effective across all RASopathies remains elusive. Further studies are required to uncover the additional mechanisms underlying this divergence, and to identify the most optimal therapeutic strategies.

## Materials and Methods

### Animals

Mice used for this study were previously described and generously provided by Mariano Barbacid and co-workers^16^. These animals express a G → T missense mutation in the second base of the twelfth codon of the endogenous *HRAS* gene locus, which replaces the normal GGA (glycine) sequence with GTA (valine) causing a constitutively active, GTP-bound HRAS^G12V^ protein. In order to be able to monitor HRAS expression, an internal ribosomal entry site–β-gal–neomycin resistance fusion protein (IRES–β-geo) cassette was inserted into the 3′ UTR of the *HRAS* gene. This allows visualization of *HRAS* gene expression patterns, by histological X-gal staining methods. *HRas* ^+*/G12V*^ mice were backcrossed (20–25 crosses) and maintained in the C57BL/6J background, and were intercrossed to generate homozygous (*HRas* ^*G12V/G12V*^), heterozygous (*HRas* ^*G12V/WT*^), and wild-type (*HRas* ^*WT/WT*^) littermates. All experiments were performed with *HRas* ^*WT/WT*^ and *HRas* ^*G12V/G12*^ mice, as more robust neurological deficits were found among homozygous *HRas* ^*G12V/G12*^ mice than in the analysis of heterozygous *HRas* ^*G12V/WT*^ mice, suggesting the existence of a gene dose effect^17^. Both males and females mice were used in all experiments.

All mice were between 8 and 14 weeks of age at the start of the behavioral experiments, housed in groups of 2–4 per cage, on a regular 12 h light/dark cycle (lights on between 7:00 am and 7:00 pm), and fed standard laboratory food *ad libitum*. Experimenters were blind to genotype and treatment. All experiments were approved by the Dutch Ethical Committee (DEC) and were in accordance with the institutional animal care and use committee guidelines.

### Western blot

To obtain hippocampal tissue, animals were anesthetized with isoflurane after which they were decapitated and tissue was quickly isolated. Tissue was homogenized in 0.1 M Tris-HCl (pH 6,8), 4% SDS supplemented with protease and phosphatase inhibitor cocktails (Sigma). The concentration of protein was adjusted to 1 μg/μl. 15 μg was used for Western blot analysis using 18-wells Criterion XT Bis-Tris precast gels (Bio-Rad). When using 26-wells gels, 10 μg protein was loaded. Primary antibodies used were HRAS (HRAS (C20), Santa Cruz Biotechnology), ERK (p44/42 MAPK, Cell Signaling), phospho-ERK (Ph-p44/42 MAPK, Cell Signaling). Actin was always included as a loading control (Actin, Millipore). Blots were probed with IR Dye secondary antibodies (IR Dye 680LT or 800CW, LI-COR) and visualized using LI-COR Odyssey infrared imager. For quantitative analysis, Odyssey V3.0 software was used.

### Immunohistochemistry

Mice were deeply anesthetized with pentobarbital and perfused transcardially with Phosphate Buffered Saline (PBS) followed by freshly prepared 4% paraformaldehyde (PFA, Sigma). Brains were carefully removed and fixed for two hours in 4% PFA, followed by 24 h incubation in 0.1 M phosphate buffer (PB) and 10% sucrose. Brains were then embedded in gelatine blocks (10% gelatine, 10% sucrose), post-fixed in 10% formaldehyde and 30% sucrose for 3 h, and kept in 30% sucrose overnight at 4 °C. The embedded brains were sectioned coronally using a freezing microtome with a section thickness of 40 μm and collected in PBS. Sections were stained with Cytochrome C or thionin, processed for LacZ staining (to visualize the expression of the HRAS gene mutation), or processed for immunohistochemistry. LacZ staining was performed by overnight incubation in 1 ml X-gal solution containing: 5% X-gal, 5 mM K3F3(CN)6, 5 mM K4F3(CN)6.3H2O, 2 mM MgCl2, 0.01% Nadeoxycholate, 0.02% NP40 in PBS. In cases where X-gal treatment was combined with immunohistochemistry, X-gal incubation preceded the other immunohistochemical steps.

For immunohistochemistry, sections were pretreated with 10 mM Na-citrate for 2 hours in 80 °C to facilitate binding of antibodies and to minimize variability introduced by the quality of perfusion. Following the blocking step, sections were incubated in primary antibody for 2 nights at 4 °C. Primary antibodies used are HRAS (HRAS (C20), Santa Cruz Biotechnology), phospho-ERK (p44/42 MAPK, Cell Signaling), ERK (p44/42 MAPK, Cell Signaling), anti-NeuN (Millipore MAB377, 1:2,000), goat anti-FoxP2 (AbCam, 1:1,000). Secondary antibodies used for confocal microscopy include FITC-conjugated donkey-anti-rabbit and Cy3-conjugated donkey-anti-sheep (Jackson Immuno Research Laboratories). DAPI (P36931, Life Technology) was used for nuclear staining. Sections stained for immunofluorescence were analyzed with Zeiss (Oberkochen, Germany) LSM700 confocal laser scanning microscope using 10x/0.45, 20x/0.8 numerical aperture objective and 40x/1.3 (oil-immersion) objectives. For immunohistochemistry, an avidin-biotin–immunoperoxidase complex method (ABC; Vector Laboratories) with diaminobenzidine (0.05%) as the chromogen and biotinylated secondary antibodies (Jackson ImmunoResearch) were used. Immunoperoxidase-stained sections were analyzed and photographed using a Leica DM-RB microscope and a Leica DC300 digital camera (Leica Microsystems GmbH, Wetzlar, Germany).

### Neuroanatomical measurements

Mice were transcardially perfused with freshly prepared 4% PFA and the brain was carefully taken out of the skull, after which the weight, height, length and width of the brain was immediately measured using a microscale (Sartorius AG, Germany) and a digital calliper (Mitutoyo Ltd., UK).

For quantititave analysis of neuronal sizes transverse serial sections were stained for cytochrome C or thionin or DAPI, NeuN, and Fox2 immunofluorescence following the procedures described above. Cross-sectional surface areas and thickness were determined using the ImageJ image analysis system. Measurements were made in matched cytochrome C-stained coronal sections (40 um thick) of WT and *HRas* ^*G12V/G12V*^ littermates. Cross sections at 3 different levels were analysed: coordinates to Bregma: 0.50 mm (Plate27), 0.02 mm (plate 31) and −0.46 mm (plate 35) according to the Paxinos and Franklin mouse brain atlas^53^. The contours of each measured cortical and corpus callosum (CC) area were traced using a manually driven cursor (Fig. 2C). Areas were calculated from the digitized data derived from a series of six sections per animal (three sections per hemisphere). Corpus callosum (CC) thickness was determined at the midline whereas thickness of the cortex was measured above the highest edge of lateral ventricle (Fig. 2C, black line in the left cortex hemisphere). The data were evaluated by unpaired t-tests to determine whether there were significant differences between genotypes.

ImageJ software was used for the analysis of cell size. Images and z-stacks were collected on a Zeiss LSM 710 confocal microscope with 10x and 40x objectives (zoom factors 1 and 0.50) from the somatosensory cortex S1 (Fig. 2F). Z-stacks were collected from cortical laminas II/III, IV/V, and VI. Each z-stack contained between 17–32 images collected with 40x objective (zoom factor 1). From each z-stack, 3 images were randomly identified for cell size analysis, without overlap between the selected images. The number of DAPI positive/NeuN negative, NeuN positive, and FoxP2 positive/NeuN positive cells were determined for each image. Cells were selected for size/area measurement according the following criteria: 1) full nucleus visible in the selected plane (DAPI), 2) cell positive for NeuN, 3) cell fully visible. An average of 20 cells per image were analysed. Contours of cells were traced manually using the Freehand selection tool of ImageJ to create regions of interest (ROI), which were subsequently analysed and saved using the ROI Manager tool of ImageJ.

### Behavioral assessment

One week long before the start of the behavioral testing, all mice were habituated to the experimentator and handling to reduce stress during experiments. For assessment of spatial learning, the Morris Water Maze (MWM) task was used as previously described^51^. Mice were handled for one minute per day, beginning one week prior to the experiment. Our water maze is a circular pool with a diameter of 1.2 meters, filled with an opaque mixture of water and white paint. Water temperature was maintained between 25–26 degrees Celsius. The escape platform has a diameter of 11 cm and is submerged 1 cm beneath the water surface. Visually-salient and readily-distinguishable distal cues were displayed on each wall of the room. Mice were trained using two 60 s trials per day, with a 30 s inter-trial interval, for five consecutive days. During training, mice were placed on the platform for 30 s and subsequently placed into the pool at pseudo-random starting positions. After reaching the platform, the mice were allowed to remain for 30 s, before returning to their home cage. If a mouse was unable to locate the platform within 60 s, the trial was concluded and the mouse was gently placed on the platform by the experimenter, and remained there for 30 s. The platform location was in a fixed position throughout all trials. For each trial, latency, distance covered, mean swim speed, and search path were measured.

Probe trials were initiated when average latencies dropped below 30 s and a significant number of mice showed selective searching. During the probe trial, the platform was removed from the pool. The mice were placed in the pool at the opposite side where the platform used to be and allowed to search for 60 s. The amount of time spent in each quadrant, and platform location crossings, were measured for each probe trial. For statistical analysis, the time in the target quadrant was compared to the average of the other three quadrants. Next, the platform was switched to another location (reversal-learning paradigm) and mice were retrained for 5 days after which memory was assessed by means of another probe trial (day 14).

### Drug treatment

Lovastatin: The HMG-CoA reductase inhibitor lovastatin was purchased from Sigma, stored according to the manufacturer’s instructions, and freshly dissolved as previously described^31^. The solution was administered by subcutaneous (SC) injection of 10 mg/kg (body weight) daily.

MEK inhibitor (PD0325901; Sigma-Aldrich) was dissolved in DMSO at a concentration of 25 mg/ml and resuspended in vehicle (0.5% hydroxypropyl methyl-cellulose with 0.2% Tween 80; Sigma-Aldrich) at a concentration of 1 mg/ml as described by^9^. The solution was administered by intraperitoneal injection (IP) of 2 mg/kg or 5 mg/kg (body weight) daily.

Lovastatin (10 mg/kg; SC), PD0325901 (2 mg/kg; IP), or vehicle (DMSO with 0.5% hydroxypropyl methyl-cellulose and 0.2% Tween 80) was injected once daily for 3 d prior to, and during, Morris water maze training 6 h prior to training. As described above, mice were trained in the hidden version of the MWM for 7 days (2 trials/day) after which the probe trial was given.

### Electrophysiology

Field and whole-cell patch-clamp recordings were performed as previously described^25^. Briefly, hippocampal slices were prepared from mice (3 to 4 week-old for whole cell recording, 3 to 6 week-old for LTD, and 3–6 week-old as well as 8–20 weeks old for LTP recordings). Mice were decapitated under deep isoflurane anesthesia, brains quickly removed and placed in ice-cold modified artificial cerebrospinal fluid (ACSF) containing (in mM): 3 KCl, 1.3 CaCl2, 2.5 MgSO4, 1.25 NaH2PO4, 10 glucose, 26 NaHCO3, and 212.3 sucrose oxygenated with 95% O2 and 5% CO2 (pH 7.3–7.4). For the whole-cell recordings transverse hippocampal slices (300 μm) were cut in ice-cold modified ACSF using a vibratome (HM650V; Microm). Slices were then stored in a holding chamber containing standard ACSF (in mM): 126 NaCl, 3 KCl, 2 MgSO4, 2 CaCl2, 10 glucose, 1.25 NaH2PO4, and 26 NaHCO3 bubbled with 95% O2 and 5% CO2 (pH 7.3) at room temperature for at least 1 h. Individual slices were transferred to the submerged recording chamber perfused with standard ACSF continuously bubbled with 95% O2 and 5% CO2 at 30–32 °C.

Whole-cell patch-clamp recordings were performed from hippocampal CA1 pyramidal neurons visualized with a Zeiss microscope using infrared video microscopy and differential interference contrast (DIC) optics. Patch electrodes were pulled from borosilicate glass capillaries and had a resistance of 3–5 MΩ when filled with intracellular solutions. Inhibitory synaptic responses were evoked using twisted platinum/iridium electrodes placed 100–150 μm from the recording site. Spontaneous inhibitory synaptic currents (IPSCs) were recorded in the presence of 6-cyano-7- nitroquinoxaline-2,3-dione (CNQX) (10 μM) and D,L-2-amino-5-phosphopentanoic acid (D,L-AP5) (20 μM) with internal solution containing (in mM): 70 K-gluconate, 70 KCl, 10 HEPES, 0.5 EGTA, 4 MgATP, 0.4 Na2GTP, 4 phosphocreatine (pH 7.3 with KOH) or 125 CsCl, 2 MgCl2, 5 NaCl, 10 HEPES, 0.2 EGTA, 4 MgATP, 0.4 Na2GTP (pH 7.3 with CsOH). Spontaneous excitatory synaptic currents (EPSCs) were recorded in the presence of picrotoxin (100 μM) and with internal solution containing (in mM): 140 K-gluconate, 1 KCl, 10 HEPES, 0.5 EGTA, 4 MgATP, 0.4 Na2GTP, 4 phosphocreatine (pH 7.3 with KOH). Miniature IPSCs/EPSCs were recorded in the presence of 1 μM TTX. Recordings were made with a patch-clamp amplifier (Multiclamp 700B; Axon Instruments, Foster City, CA, USA). Signals were low-pass filtered at 2–4 KHz and digitized at 10 KHz. Series resistance was constantly monitored, and the cells were rejected from analysis if the resistance changed by >15%. No series resistance compensation was used. Only cells with stable resting membrane potential and overshooting action potentials with stable amplitude were included in the study. Resting membrane potential was measured in bridge mode (I = 0) immediately after obtaining whole-cell access.

Field EPSP recordings were performed as previously described^25, 52^. Briefly, field excitatory postsynaptic potentials (fEPSPs) were evoked by stimulating the Schaffer collateral/commissural pathway with a bipolar twisted platinum/iridium electrode (FHC, Bowdoinham, ME). Baseline stimulation (100-μsec duration) was adjusted to 30–40% (LTP) or 50% (LTD) of the maximum fEPSP amplitude. LTP was induced using different LTP protocols including: high frequency stimulation (100 Hz, 1 s) and theta burst stimulation (TBS) protocols. The TBS protocol consists of four trains at 5 Hz, each composed of four pulses at 100 Hz. Chemical mGluR-dependent LTD was evoked by application of (RS)-3,5-dihydroxyphenylglycine (DHPG, 100 μM) for 5 min. For LTD rescue experiments, either MEK-inhibitor (U0126, Tocris Bioscience, 1 μM) or DMSO was added to the recording solution before starting the protocol. For NMDA-dependent LTD, the average LTP or LTD over the last 10 minutes of each measurement was used for analysis.

### Data and statistical analysis

Spontaneous synaptic currents (IPSCs and EPSCs) data were analyzed with Mini Analysis (Synaptosoft Inc., Decatur, GA). All events were detected with a threshold >3× root-mean-square (RMS) of baseline noise. The detected currents were manually inspected to exclude false events. Passive and active membrane properties were analyzed offline using Clampfit 10 (Axon Instruments). Changes in synaptic strength were quantified for statistical comparisons by normalizing and averaging fEPSP slopes during the last 10 min of experiments relative to baseline.

All data are reported as mean ± SEM. Cumulative miniature and spontaneous synaptic current amplitude and inter-event interval (IEI) distributions were analyzed for statistical significance using the Kolmogorov-Smirnov (K-S) test (Statistica), and a conservative critical probability level of *P* < 0.01. All other statistical tests, including t-tests and one- or two-way (M)ANOVAs ([multivariate] analysis of variance), were performed using a critical probability of *P* < 0.05 and two-sided testing (SPSS version 20.0). *Post-hoc* Bonferroni correction analysis was performed only when an ANOVA yielded a significant (*P* < 0.05) main effect. Variance within each genotype or treatment was visualized in graphs. There were no overt differences in the variances between groups. All distributions were normal, either with 95% or 99% of confidence, and fulfilled the criteria for parametric testing. To detect or rule out meaningful, statistically significant differences between genotypes, we intended to include 10–15 animals per genotype for behavioral experiments, 5–10 animals per genotype for biochemical experiments, and 10–20 cells per genotype for electrophysiological measures. In general, this allowed us to reliably detect effect sizes of >15%. Genotypes were unblinded only after analysis was completed. Randomization of animals was exclusively implemented by the only person who had access to the genotypes (M.E.), and was typically based on the date of birth and the random distribution of mutant and wild-type mice in each litter. *P* < 0.05 is indicated with one asterisk (*), *P* < 0.01 with two asterisks (**), and *P* < 0.001 with three asterisks (***). P values ≥ 0.05 were considered as not rejecting the null hypothesis, and reported as not significant (N.S.).
